# Formation of a mixed-valence Cu(i)/Cu(ii) metal–organic framework with the full light spectrum and high selectivity of CO_2_ photoreduction into CH_4_†

**DOI:** 10.1039/d0sc03754k

**Published:** 2020-09-02

**Authors:** Yajun Gao, Lei Zhang, Yuming Gu, Wenwei Zhang, Yi Pan, Weihai Fang, Jing Ma, Ya-Qian Lan, Junfeng Bai

**Affiliations:** State Key Laboratory of Coordination Chemistry, School of Chemistry and Chemical Engineering, Nanjing University Nanjing 210023 China; School of Chemistry and Chemical Engineering, Shaanxi Normal University Xi'an 710119 China bjunfeng@nju.edu.cn bjunfeng@snnu.edu.cn; Key Laboratory of Mesoscopic Chemistry of Ministry of Education, School of Chemistry and Chemical Engineering, Nanjing University Nanjing 210023 China majing@nju.edu.cn; Jiangsu Collaborative Innovation Centre of Biomedical Functional Materials, Jiangsu Key Laboratory of New Power Batteries, School of Chemistry and Materials Science, Nanjing Normal University Nanjing 210023 China yqlan@njnu.edu.cn

## Abstract

Based upon the hetero-N,O ligand of pyrimidine-5-carboxylic acid (Hpmc), a new semiconductive Cu(i)/Cu(ii) mixed-valence MOF with the full light spectrum and a novel topology of {4^3^·6^12^·8^6^}_2_{4^3^·6^3^}_2_{6^3^}_6_{6^4^·8^2^}_3_, {(Cu_4_I_4_)_2.5_[Cu_3_(μ_4_-O) (μ_3_-I) (pmc)_3_(Dabco)_3_]·2.5DMF·2MeCN}_∞_ (NJU-Bai61, NJU-Bai for Nanjing University Bai group; Dabco = 1,4-diazabicyclo [2.2.2] octane), was synthesized stepwise. NJU-Bai61 exhibits good water/pH stabilities and a relatively large CO_2_ adsorption capacity (29.82 cm^3^ g^−1^ at 1 atm, 273 K) and could photocatalyze the reduction of CO_2_ into CH_4_ without additional photosensitizers and cocatalysts and with a high CH_4_ production rate (15.75 μmol g^−1^ h^−1^) and a CH_4_ selectivity of 72.8%. The CH_4_ selectivity is the highest among the reported MOFs in aqueous solution. Experimental data and theoretical calculations further revealed that the Cu_4_I_4_ cluster may adsorb light to generate photoelectrons and transfer them to its Cu_3_OI(CO_2_)_3_ cluster, and the Cu_3_OI(CO_2_)_3_ cluster could provide active sites to adsorb and reduce CO_2_ and deliver sufficient electrons for CO_2_ to produce CH_4_. This is the first time that the old Cu(i)_*x*_X_*y*_L_*z*_ coordination polymers' application has been extended for the photoreduction of CO_2_ to CH_4_ and this opens up a new platform for the effective photoreduction of CO_2_ to CH_4_.

## Abstract 2

A Cu(i)/Cu(ii) MOF with a full light spectrum was synthesized, exhibiting a photocatalytic CO_2_-to-CH_4_ selectivity of 72.8% in aqueous solution.

## Graphical abstract

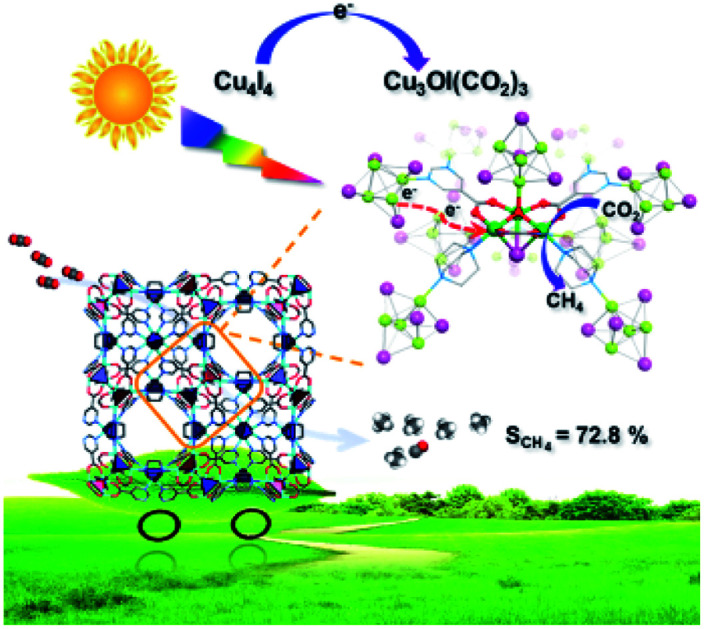

## Introduction

Due to climate change, CO_2_ capture and conversion has recently, become one of the greatest concerns.^1^ In particular, the photoreduction of CO_2_ into value-added chemicals (such as CO, HCOOH, CH_4_, and so on) has attracted great attention, because it can be considered as a promising approach for solar-to-chemical energy conversion by mimicking the natural photosynthetic process to achieve a carbon neutral economy.^2^ In the past few decades, diverse photocatalysts have been extensively employed for the photocatalytic CO_2_ reduction reaction (CO_2_RR).^3^ Homogeneous/molecular catalysts exhibit high selectivity and efficiency, but low activity due to catalyst deactivation,^4^ whereas heterogeneous/inorganic catalysts show high activity and efficiency, but low selectivity.^5^ Very recently, due to their high surface area, inorganic–organic hybrid nature, structural and functional diversity and tunability, metal–organic frameworks (MOFs) may combine the advantages of the traditional homogeneous/heterogeneous catalysts and are emerging as promising platforms for the photocatalytic CO_2_RR.^6^

Since 2011,^7^ many MOFs have been designed for the photocatalytic CO_2_RR targeting to improve their efficiency, activity and selectivity by functionalizing organic ligands, optimizing metal ions/clusters, and making MOF-based composites.^8^ Although, some achievements have been made, research on MOF-based photocatalysts to date is still in its early stages. In terms of the reductive products, most reported MOFs predominantly produce the 2e^−^/2H^+^ products of CO/HCOOH.^8*a*,9^ Due to the fact that the photocatalytic reduction of CO_2_ into CH_4_ is more difficult than with other C1 fuels, because it involves a complex 8e^−^/8H^+^ reduction process, *i.e.*, multiple steps of hydrogenation and deoxygenation reactions, and requiring the highest kinetic barrier of up to 818.3 kJ mol^−1^,^10^ the reported MOF catalysts capable of producing even low or moderate yields of CH_4_ are still rare. Thus, design of MOFs with high selectivity for the reduction of CO_2_ into CH_4_ is a great challenge.^11^

The Cu(i)_*x*_X_*y*_L_*z*_ (where X = Cl, Br or I; L = N, P or S containing organic ligands) are almost the oldest coordination polymers with diversified structures and interesting properties, such as luminescence and semiconductivity, and so on.^12^ Very recently, their use has been demonstrated for photocatalytic H_2_ evolution.^13^ Herein the exploration of these polymers as promising platforms for CO_2_ capture and conversion is reported. From a simple hetero-N,O ligand pyrimidine-5-carboxylic acid, a Cu_4_I_4_ and Cu_3_OI(CO_2_)_3_ cluster based and semiconductive Cu(i)/Cu(ii) mixed-valence MOF (**NJU-Bai61**) with a full light spectrum, which exhibits good water and pH stabilities and the relatively large CO_2_ adsorption capacity (29.82 cm^3^ g^−1^ at 1 atm, 273 K) was successfully constructed. In addition, **NJU-Bai61** could photocatalyze the reduction of CO_2_ into CH_4_ without additional photosensitizers and cocatalysts and with a high CH_4_ production (15.75 μmol g^−1^ h^−1^) and CH_4_ selectivity of 72.8%. As far as is known, the CH_4_ selectivity is the highest among the reported MOFs in the aqueous solution. Upon light irradiation, its Cu_4_I_4_ clusters as photoelectron generators could transfer photoelectrons to the Cu_3_OI(CO_2_)_3_ clusters, whereas the Cu_3_OI(CO_2_)_3_ clusters could provide active sites for adsorbing and reducing CO_2_ and act as photoelectron collectors for delivering enough electrons to CO_2_ for CH_4_ evolution.

## Results and discussion

From CuI and the Hpmc ligand and using Dabco as the structural directing agent, like many Cu(i)_*x*_X_*y*_L_*z*_, a Cu_4_I_4_ cluster-based copper(i) coordination polymer, {(Cu_4_I_4_) (Hpmc)_2_}_∞_ (**NJU-Bai61p**) was initially obtained. **NJU-Bai61p** is a 2D layered and 4-connected network with sql topology (Fig. S3, ESI†), in which each Hpmc ligand uses its N-donor center to link to a 4-coordinated Cu(i) in a tetrahedral coordination geometry resulting in a [Cu_4_I_4_N_4_] moiety, leaving its COOH functional group uncoordinated (Fig. S4, ESI†).

**Scheme 1:**
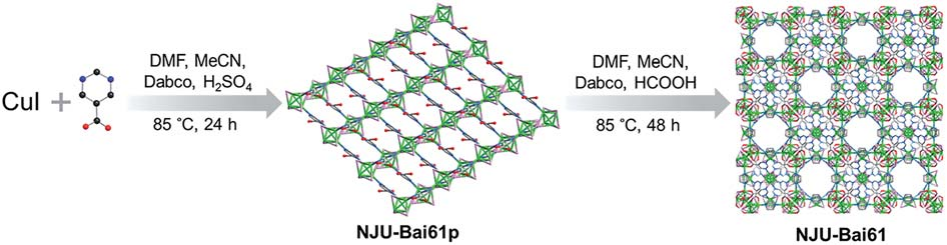
A schematic view of the preparation of **NJU-Bai61**.

Later on, by changing the acid and extending the time, **NJU-Bai61p** was further transformed into **NJU-Bai61** (Scheme 1). Compared with **NJU-Bai61p**, the Hpmc ligands in **NJU-Bai61** were deprotonated, coordinated with Cu(ii) ions in a bridging bidentate mode, facilitating the formation of the Cu_3_OI(CO_2_)_3_ cluster. The Cu_3_OI(CO_2_)_3_ cluster is 7-connected and surrounded by one Cu_4_I_4_ cluster, three pmc and three Dabco auxiliary ligands. All the Cu(ii) ions in this new cluster adopt 5-coordinated geometry with two O atoms from two independent pmc linkers, one N atom from the Dabco linker, one μ_3_-I^−^ ion shared by three Cu(ii) ions, and one μ_4_-O^2−^ ion shared by three Cu(ii) ions and one Cu(i) ion from the Cu_4_I_4_ cluster (Fig. S6, ESI†). Remarkably, the Cu_4_I_4_ clusters in **NJU-Bai61** exist in two different coordination environments. One is the same as that of **NJU-Bai61p** and can form a 4-connected [Cu_4_I_4_N_4_] moiety, whereas the other is the Cu_4_I_4_ cluster which is linked by three N atoms from three Dabco ligands and one μ_4_-O^2−^ ion to form a 4-connected [Cu_4_I_4_N_3_O] moiety (Fig. S5, ESI†).

**Fig. 1:**
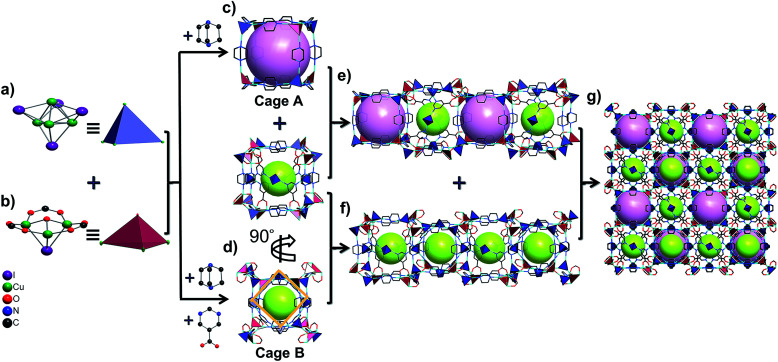
(a) and (b) Cu_4_I_4_ and Cu_3_OI(CO_2_)_3_ clusters are illustrated by two types of tetrahedrons; (c) and (d) two types of cubic cages in **NJU-Bai61**: cage A, lavender; cage B, lime; (e) the 1D channel consists of the cages A and B; (f) the 1D cage-stacked chain consists of cages B; (g) the 3D framework of **NJU-Bai61** with the 1D channels and chains.

Furthermore, these Cu_4_I_4_ and Cu_3_OI(CO_2_)_3_ clusters are bridged by pmc and Dabco ligands to form two types of cubic cages. The larger one (cage A) is composed of four Cu_4_I_4_ clusters and four Cu_3_OI(CO_2_)_3_ clusters arranged alternately as vertices and 12 linear Dabco ligands as edges with a diameter of about 8.0 Å (Fig. 1c). The smaller one (cage B) is composed of eight pairs of [Cu_4_I_4_–Cu_3_OI(CO_2_)_3_] linkage clusters as vertices and 12 Dabco ligands as edges, in which there exists a square with a diameter of about 6.4 Å based on four pmc linkers and Cu_4_I_4_ clusters located at the center of the four facets of this cage (Fig. 1d and S7, ESI†). The cages A and B connect alternately with each other to form a 1D channel by sharing quadrilateral windows, whereas the B cages connect with each other to form a 1D cage-stacked chain by sharing the facets including a quadrilateral window and a Cu_4_I_4_ cluster (Fig. 1e, f, and S8, ESI†). Therefore, these 1D channels and chains are arranged in an alternating fashion to form a 3D porous framework based on the cages A and B ratio of 1 : 3, in which each cage A shares facets with six cage Bs and each cage B shares facets with two cage As and four cage Bs (Fig. 1g and S9, ESI†). From the viewpoint of structural topology, pmc ligands, Cu_4_I_4_ and Cu_3_OI(CO_2_)_3_ clusters could be regarded as 3-connected triangular nodes, 4-connected tetrahedral nodes, and 7-connected single cap octahedron nodes, respectively. Consequently, **NJU-Bai61** is a new (3,4,4,7)-connected network with the point symbol {4^3^·6^12^·8^6^}_2_{4^3^·6^3^}_2_{6^3^}_6_{6^4^·8^2^}_3_ (Fig. S10, ESI†).

The phase purities and thermal stabilities of **NJU-Bai61p** and **NJU-Bai61** were confirmed using PXRD and TG analyses (Fig. S13 and S14, ESI†). As shown in Fig. S15–S17 (ESI†), they are quite stable under water and other organic solvents. Furthermore, they are also stable under the broad variation of the pH values.

**Fig. 2:**
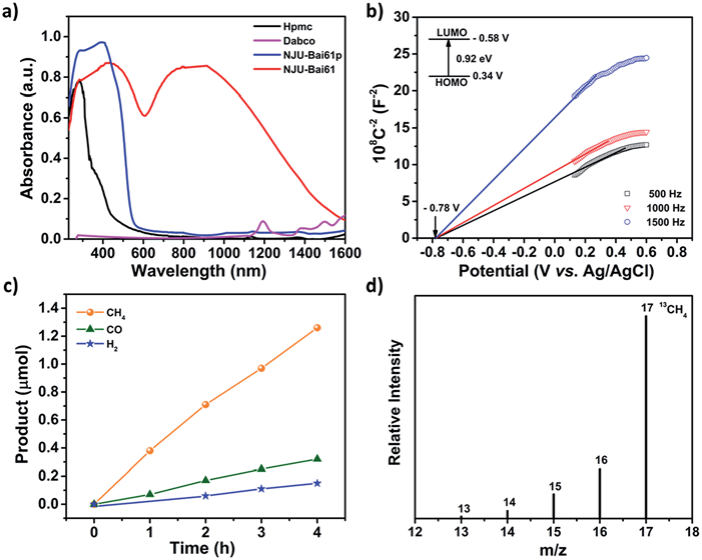
(a) The UV-Vis-NIR absorption spectra of **NJU-Bai61p** and **NJU-Bai61**; (b) Mott–Schottky plots for **NJU-Bai61**; (c) the amounts of CH_4_, CO and H_2_ produced as a function of the irradiation time over **NJU-Bai61**; (d) the mass spectral analysis of ^13^CH_4_ recorded under a ^13^CO_2_ atmosphere using **NJU-Bai61** as the catalyst.

**NJU-Bai61p** exhibits a visible light adsorption up to 550 nm due to the Cu_4_I_4_ cluster to linker charge transfer (CLCT) transition (Fig. 2a and Table S2, ESI†). Very interestingly, **NJU-Bai61** shows the widest absorption band among the reported MOFs with the edge up to 1400 nm, which are mainly dominated by intra metal cluster transfer (ICT), CLCT, and metal cluster-to-metal cluster charge transfer (CCCT) transitions (Fig. 2a and Table S3, ESI†). The bandgaps of semiconductive **NJU-Bai61p** and **NJU-Bai61** were estimated to be 2.33 eV and 0.92 eV, respectively, (Fig. S18, ESI†), which could be correlated with the calculated HOMO–LUMO gaps of 2.16 eV and 1.25 eV for the corresponding cluster models, respectively, (Tables S4 and S5, ESI†). The solid state of **NJU-Bai61** with a periodic boundary condition (PBC) model for the band gap was further calculated, showing a narrow band gap of 0.65 eV (Fig. S19, ESI†). The Mott–Schottky measurements further revealed that they were typical n-type semiconductors and their conduction bands (CB) were −0.55 V and −0.58 V, which were more negative than the reduction potentials for the conversion of CO_2_ to CO and CH_4_ (Fig. 2b and S20, ESI†).^8*a*^ Thus, they are very promising for the CO_2_ photoreduction applications.

The photocatalytic reduction of CO_2_ over the activated **NJU-Bai61** was further investigated. The amount of CH_4_ was 1.26 μmol (*i.e.*, 15.75 μmol g^−1^ h^−1^) after 4 h. Except for the small amounts of CO (0.32 μmol, *i.e.*, 4 μmol g^−1^ h^−1^) and H_2_ (0.15 μmol, *i.e.*, 1.87 μmol g^−1^ h^−1^), no other products, such as HCOOH, CH_3_OH and HCHO, were detected (Fig. 2c, S22 and S23, ESI†). The **NJU-Bai61** exhibited a CH_4_ selectivity of 72.8% in aqueous solution, which was the highest among the reported MOFs (Table S8, ESI†). No obvious change of the CH_4_ activity occurred during the four continuous runs (Fig. S24, ESI†). The XRD patterns obtained before and after its photocatalytic experiments revealed the structural robustness of the catalyst (Fig. S27, ESI†). The isotopic ^13^CO_2_ tracing experiment was also performed to confirm that the carbon source of CH_4_ did indeed come from the used CO_2_ rather than the degradation of organics in the reaction (Fig. 2d).^11*b*^ For comparison, the use of **NJU-Bai61p** as the photocatalyst was also investigated under the same conditions and only CO (1.37 μmol, *i.e.*, 17.13 μmol g^−1^ h^−1^) and H_2_ (1.34 μmol, *i.e.*, 16.75 μmol g^−1^ h^−1^) were detected after 4 h (Fig. S25, ESI†). This result may reveal that Cu_3_OI(CO_2_)_3_ clusters in **NJU-Bai61** could provide active sites for CH_4_ evolution.

Then in-depth research was carried out to discover the reason underlying the high efficiency of CH_4_ evolution. As for **NJU-Bai61**, the BET surface area was 248.1 m^2^ g^−1^ and the CO_2_ uptakes at 273 K and 298 K were 29.82 and 19.69 cm^3^ g^−1^, respectively, which was helpful for the subsequent CO_2_ conversion (Fig. S28–S30, ESI†). The electrostatic potential analysis may further reveal that the Cu(ii) centers in Cu_3_OI(CO_2_)_3_ clusters are the most favorable sites for the nucleophilic attack of CO_2_ (Fig. S31, ESI†). The local interactions between Cu(ii) sites and CO_2_ molecules were investigated using the *in situ* FTIR technology. The adsorption of CO_2_ onto the Cu(ii) sites in **NJU-Bai61** was a 16 cm^−1^ red shift of the asymmetric stretching mode of CO_2_ (*ν* = 2359 cm^−1^), indicating the stronger binding between the CO_2_ and Cu(ii) sites (Fig. S33, ESI†).^11*b*^ However, for **NJU-Bai61p**, no shift existed after CO_2_ adsorption (Fig. S32, ESI†). Moreover, this experimental phenomenon was explained by the DFT calculations in which the peaks were also red-shifted and the adsorbed CO_2_ molecule takes a slightly bent geometry to facilitate the CO_2_ activation (Fig. S34 and Table S9, ESI†).^14^ Furthermore, its fluorescence was quenched in comparison to **NJU-Bai61p**, indicating that the photo-excited electrons of the Cu_4_I_4_ clusters were transferred to the Cu_3_OI(CO_2_)_3_ clusters, making it act as a photoelectron collector to provide electrons for the adsorbed CO_2_ (Fig. S35, ESI†).

**Fig. 3:**
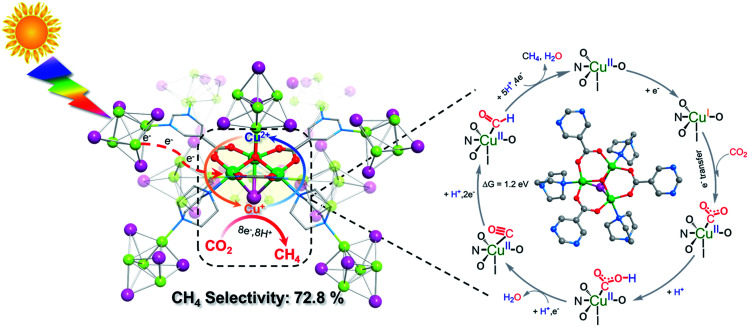
A proposed reaction pathway together with free energy difference (Δ*G*) for the photocatalytic CO_2_-to-CH_4_ conversion over **NJU-Bai61**.

An energetically feasible reaction pathway was calculated using DFT with the relative free energy, Δ*G*, for each step shown in Fig. 3 and S38 (ESI).† Upon light irradiation, the Cu_4_I_4_ clusters in **NJU-Bai61** may adsorb light to generate the photoelectrons and transfer them to the Cu_3_OI(CO_2_)_3_ clusters, whereas the Cu_3_OI(CO_2_)_3_ clusters could supply electrons to the adsorbed CO_2_ for CH_4_ evolution. In the first step, the adsorbed CO_2_ molecule accepted an electron and a proton to generate the COOH*. Then the COOH* combines with the second electron–proton pair to generate CO*. The CO* was reduced to the CHO* by accepting two electrons and a proton, and further combined with a total of four electrons and five protons to generate CH_4_. In the photocatalytic process, the Cu_4_I_4_ cluster could serve as a photosensitizer and donated the energy of 2.16 eV to the conversion process of CO* to CHO* at the Cu_3_OI(CO_2_)_3_ cluster which was an endothermic process with the Δ*G* of 1.2 eV. Moreover, the stronger CO binding affinity on **NJU-Bai61** (*E*_b_ = −20.13 eV) in comparison with that on only Cu(i)-contained **NJU-Bai61p** (*E*_b_ = −8.05 eV) may further stabilize the CO@Cu_3_IO(CO_2_)_3_ complex to complete the CO_2_-to-CH_4_ conversion (Fig. S39, ESI†).

## Conclusions

In summary, a novel Cu_4_I_4_ and Cu_3_OI(CO_2_)_3_ cluster based and semiconductive Cu(i)/Cu(ii) mixed-valence MOF with the full light spectrum, NJU-Bai61, was successfully produced, which exhibits good water stability, pH stability and a relatively large CO_2_ adsorption capacity. NJU-Bai61 could photocatalyze the reduction of CO_2_ into CH_4_, without additional photosensitizers and cocatalysts, but with a high CH_4_ production and significantly high CH_4_ selectivity of 72.8% (the highest among the reported MOFs in aqueous solution). It was revealed that the Cu_4_I_4_ and Cu_3_OI(CO_2_)_3_ clusters may play the role of photoelectron generators and collectors, respectively. This work firstly expands the old Cu(i)_*x*_X_*y*_L_*z*_ coordination polymers' application into the reduction of CO_2_ to CH_4_ and may open up a new system of MOFs for the reduction of CO_2_ to CH_4_ with high selectivity.

## Experimental section

### Synthesis of **NJU-Bai61p**

A mixture of Hpmc (11 mg, 0.09 mmol), CuI (30 mg, 0.16 mmol), Dabco (6 mg, 0.05 mmol), H_2_SO_4_ (10 μL), DMF (1.0 mL), and MeCN (3.0 mL) was sealed in a 20 mL Pyrex tube and kept in an oven at 85 °C for 1 day. After washing with DMF, yellow block crystals were obtained. Yield: 2.5 mg (6%). Selected IR (cm^−1^): 3036, 2666, 2554, 1713, 1586, 1441, 1398, 1330, 1297, 1202, 1170, 1119, 1090, 1054, 996, 908, 837, 749, 695, 667, 568. Elemental analysis (%) calcd. for Cu_2_I_2_C_5_H_4_N_2_O_2_: C 11.89, H 0.80, N 5.54; found: C 11.96, H 1.00, N 5.52.

### Synthesis of **NJU-Bai61**

A single crystal of **NJU-Bai61p** (10 mg), Dabco (4 mg, 0.036 mmol) and CuI (20 mg, 0.11 mmol) were added to 1.0 mL of DMF and 3.0 mL of MeCN. To this was added 60 μL of HCOOH with stirring. The mixture was sealed in a Pyrex tube and heated to 85 °C for 2 d. Dark-red octahedral crystals were obtained and further characterized by PXRD and the results are shown in Fig. S1 (ESI†). Yield: 8.8 mg (25%). Selected IR (cm^−1^): 3392, 3108, 2952, 2883, 2840, 1681, 1652, 1587, 1435, 1377, 1319, 1218, 1170, 1087, 1050, 1000, 924, 840, 805, 764, 700, 612, 583, 468, 420. Elemental analysis (%) calcd. for Cu_13_I_11_C_44.5_H_68.5_N_16.5_O_9.5_: C 16.66, H 2.15, N 7.20; found: C 16.87, H 2.30, N 6.98.

### Sample activation

The as-synthesized sample of **NJU-Bai61** was soaked in MeOH for 5 d with refreshing of the MeOH every 8 h. Then, the solvent-exchanged sample was activated at 70 °C and under vacuum for 10 h to obtain the activated **NJU-Bai61**.

### Photocatalytic reaction

The photocatalytic CO_2_ reduction experiments were carried out on an evaluation system (CEL-SPH2N, CEAULIGHT, China), in a 100 mL quartz container. A 300 W xenon arc lamp (300 < λ < 2500 nm) was utilized as the irradiation source. The 20 mg MOFs (**NJU-Bai61p** or the activated **NJU-Bai61**) were dispersed in 50 mL of a solution of triethylamine and water (TEA/H_2_O = 5 : 45 *v/v*). The suspension was pre-degassed with CO_2_ (99.999%) for 30 min to remove the air before irradiation. The reaction was stirred constantly with a magnetic bar to ensure the photocatalyst particles remained in suspension. The temperature of the reaction was maintained at 25 °C by a circulating cooling water system. The gaseous product was measured by gas chromatography (GC-7900, CEAULIGHT, China) with a flame ionization detector (FID) and a thermal conductivity detector (TCD). An ion chromatography (LC-2010 Plus, Shimadzu, Japan) was used for the detection of HCOO^−^. The concentration of Cu in the solution before and after catalysis was determined using an ICP-OES system (Optima 5300 DV, PerkinElmer). Before the photocatalytic reaction, the suspension of the activated **NJU-Bai61** (220 mg), TEA (5 mL) and H_2_O (45 mL) was pre-degassed with CO_2_ (99.999%) for 30 min to remove the air, then 2 mL of the filtrate was removed and a Cu concentration of 0.6 mg L^−1^ was detected. Thus, the concentration of dissolved Cu ions of the activated **NJU-Bai61** was 0.05% before catalysis. After the photocatalytic reaction, 2 mL of filtrate was also removed and the concentration of Cu in the filtrate was determined to be 13.8 mg L^−1^. Thus, the concentration of dissolved Cu ions of the activated **NJU-Bai61** was 1.1%. The cycling experiment was carried out as follows: at the end of each run, the suspension was centrifuged and the supernatant was removed. Then the recovered catalyst was washed with distilled water and dried in air at 60 °C before the next cycle.

## Conflicts of interest

There are no conflicts to declare.

## Supplementary Material

SC-011-D0SC03754K-s001

SC-011-D0SC03754K-s002
