# Supplementary material for: Formation of a mixed-valence Cu(i)/Cu(ii) metal–organic framework with the full light spectrum and high selectivity of CO2 photoreduction into CH4
Source: Chem Sci. 2020 Sep 2;11(37):10143–8. doi: 10.1039/d0sc03754k (PMC8162430; doi:10.1039/d0sc03754k)
Supplement: SC-011-D0SC03754K-s001 [file SC-011-D0SC03754K-s001.pdf]

Electronic Supplementary Material (ESI) for Chemical Science.
This journal is © The Royal Society of Chemistry 2020

Formation of a Mixed-Valence Cu(I)/Cu(II) Metal-Organic Framework with the Full Light Spectrum and High Selectivity of CO_2 Photoreduction into CH_4

Yajun Gao[a+], Lei Zhang[d+], Yuming Gu[c+], Wenwei Zhang[a], Yi Pan[a], Weihai Fang[c], Jing Ma[*c], Ya-Qian Lan[*d], and Junfeng Bai[*a,b]

[a] State Key Laboratory of Coordination Chemistry, School of Chemistry and Chemical Engineering, Nanjing University, Nanjing 210023, China

[b] School of Chemistry and Chemical Engineering, Shaanxi Normal University, Xi'an 710119, China

[c] Key Laboratory of Mesoscopic Chemistry of Ministry of Education, School of Chemistry and Chemical Engineering, Nanjing University, Nanjing 210023, China

[d] Jiangsu Collaborative Innovation Centre of Biomedical Functional Materials, Jiangsu Key Laboratory of New Power Batteries, School of Chemistry and Materials Science, Nanjing Normal University, Nanjing 210023, China

*Email: bjunfeng@nju.edu.cn; bjunfeng@snnu.edu.cn (J.B.).

*E-mail: majing@nju.edu.cn (J.M.).

*Email: yqlan@njnu.edu.cn (Y.-Q.L.).

Contents

Section 1. Experimental sections …….S2

Section 2. Computational details…….S4

Section 3. Single-crystal X-ray structure determination…….S4

Section 4. IR and XPS analyses…….S10

Section 5. Stability tests…….S12

Section 6. Characterization of band structure…….S17

Section 7. Electrical conductivity tests…….S24

Section 8. The research of photocatalytic CO_2 reduction reaction…….S25

Section 9. The research of photocatalytic pathway of CO_2-to-CH_4 reduction…….S30

References…….S37

Section 1. Experimental sections

Materials and general methods. All chemical reagents were obtained from commercial sources and, unless otherwise noted, were used as received without further purification. Elemental analyses (C, H, and N) were performed on a Perkin-Elmer 240 analyzer. The IR spectra were obtained in the 4000~400 cm^{-1} on a VECTOR TM 22 spectrometer using KBr pellets. The in situ FTIR experiments were performed in an IR cell made of quartz on the PerkinElmer Frontier FT-IR Spectrometer with the MCT detector. Thermal gravimetric (TG) analyses were performed under N_2 atmosphere (100 ml min^{-1}) with a heating rate of 10 °C min^{-1} using a 2960 SDT thermogravimetric analyzer. Powder X-ray diffraction (PXRD) data were collected on a Bruker D8 ADVANCE X-ray diffractometer with Cu/Kα radiation. X-ray photoelectron spectroscopy (XPS) was used a ULVAC - PHI 5000 VersaProbe with an Al Kα microfocused X-ray source and the C1s peak at 284.8 eV as internal standard. UV-Vis-NIR absorption spectra were recorded in diffuse reflectance mode on a UV-3600 Shimadzu spectrometer. The optical band gap (E_g) of samples calculation based on UV-Vis diffusion spectra could be estimated following the equation proposed by Tauc, Davis, and Mott: $(\alpha h\nu)^2 = h\nu - E_g$ (Where α stands for absorption coefficient, h is Planck's constant and ν represents frequency of vibration)[1]. Photoluminescence (PL) spectra were measured on a Hitachi F-4600 photoluminescence spectrophotometer. The decay lifetime was measured on an Edinburgh Instruments FLS 980 fluorescence spectrometer. The ^1H NMR spectra were recorded on a Bruker DRX-500 spectrometer with tetramethylsilane as an internal reference. The ^{13}C NMR spectra were measured using a chromatography-mass spectrometry (7890A and 5975C, Agilent).

1.1 Conductivity tests

The conductivity of the samples was obtained from Keithley 2400 source meter on CRX-4K High Performance Closed Cycle Refrigerator-based Probe Station at room temperature. The sample powders were pressed under pressure of 10 Mpa into pellets using the conductive carbon adhesive contacts with the "two-probe method" for the current-voltage (I-V) measurements in -10 to 10 V. The electrical conductivity σ can be expressed as, $\sigma = G\cdot L/A$, where L and G are the length, electrical conductance of the pellet, respectively, and A is the area of the conductive carbon adhesive.

1.2 Electrochemical measurements

The Mott-Schottky measurements were performed on a three-electrode electrochemical workstation CHI 660E (CH Instruments, USA) at frequencies of 500, 1000 and 1500 Hz. Preparation of

the working electrode: 2 mg samples were dispersed in a mixed solution of 990 μL ethanol and 10 μL Nafion D-520 dispersion solutions to generate the homogeneous slurry. Subsequently, 200 μL of slurry was transferred and coated on fluoride-tin oxide (FTO) glass plates (1 cm × 2 cm) then dried at room temperature. The Ag/AgCl electrode was employed as the reference electrode and platinum plate was used as the counter electrode, respectively. A 0.2 M of Na_2SO_4 solution was used as the electrolyte.

The transient photocurrent responses were carried out under light irradiation conditions (300 W xenon arc lamp, CEL-HXF300/CEL-HXUV300, 200 mW/cm^2). The preparation of working electrode was the same as above, but the electrolyte was instead of 0.5 M Na_2SO_4 aqueous solution.

1.3 Gas Sorption Measurements.

Low-pressure adsorption isotherms of N_2 (99.999%) and CO_2 (99.999%) were performed on Quantachrome Autosorb IQ-2 surface area and pore size analyzer. Before analysis, about 100 mg samples were activated by using the "outgas" function of the surface area analyzer. Helium (99.999%) was used for the estimation of the free space (warm and cold), assuming that it was not adsorbed at any of the studied temperatures. The specific surface area was determined using the Brunauer-Emmett-Teller (BET) and the Langmuir equation from the N_2 sorption data at 77 K. When applying the BET theory, we made sure that our analysis satisfied the two consistency criteria as detailed by Walton and co-workers.[2]

Calculations of isosteric heat of adsorption (Q_{st}). A virial-type[3] expression comprising the temperature-independent parameters a_i and b_j was employed to calculate the enthalpies of adsorption for CO_2 (at 273 and 298 K) on **NJU-Bai61**. In each case, the data were fitted using the equation:

$$\ln P = \ln N + 1/T \sum_{i=0}^{m} a_i N^i + \sum_{j=0}^{n} b_j N^j \qquad (1)$$

Here, P is the pressure expressed in Torr, N is the amount adsorbed in mmol g^{-1}, T is the temperature in K, a_i and b_j are virial coefficients, and m, n represent the number of coefficients required to adequately describe the isotherms (m and n were gradually increased until the contribution of extra added a and b coefficients was deemed to be statistically insignificant towards the overall fit, and the average value of the squared deviations from the experimental values was minimized). The values of the virial coefficients a_0 through a_m were then used to calculate the isosteric heat of adsorption using the following expression.

$$Q_{st} = -R \sum_{i=0}^{m} a_i N^i \qquad (2)$$

Q_{st} is the coverage-dependent isosteric heat of adsorption and R is the universal gas constant. The heat of CO_2 adsorption for **NJU-Bai61** in the manuscript are determined by using the adsorption data measured in the pressure range from 0 ~ 1 bar (273 and 298 K), which is fitted by the virial equation very well ($R^2 > 0.9999$).

Section 2. Computational details

All the calculations were performed with the Gaussian 16 package suite.[4] Geometry optimizations were carried out by using density functional theory (DFT) with the B3LYP functional.[5] The 6-31G(d) basis set was employed for C, H, O, and N atoms, while the LANL2DZ basis set, together with the related effective core potentials,[6] was used for Cu and I atoms. The time-dependent DFT (TDDFT) calculations for the vertical excitation energies of ligands or MOF units were performed at the optimized geometries for them in the ground states. The emission energies of Cu_4I_4 clusters were carried out at the TDDFT optimized geometries in the first excited singlet (S_1) states. The vibrational analysis was performed to test the optimized stationary points, and to calculate the free energies along reaction pathways of the photocatalytic CO_2-to-CH_4 conversion at the $Cu_3OI(CO_2)_3$ cluster in **NJU-Bai61**. The binding energy (E_b) was obtained by calculating the energy difference between the total energy of the complex system (E_{sys}) and the sum of individual energy of the MOF (E_{MOF}) and CO (E_{CO}), respectively, which was expressed as：

$$E_b = E_{sys} - E_{MOF} - E_{CO} \qquad (3)$$

To understand the fundamental electronic structures of NJU-Bai61, band structure and density of states (DOS) calculations were performed by density functional theory (DFT), as implemented in Cambridge Serial Total Energy Package (CASTEP)[7] module in the Materials Studio software package.[8] The generalized gradient approximation (GGA) in the Perdew–Burke–Ernzerhof (PBE)[9] forms was employed, and Grimme method[10,11] was applied to take the van der Waals interaction into consideration. In Figure X2, NJU-Bai61 shows a narrow band gap of 0.65 eV, which is agreement with the experimental results, indicating that it can absorb the light for the catalytic reaction from CO_2 to CH_4.

Section 3. Single-crystal X-ray structure determination

Single-crystal X-ray diffraction data were measured on a Bruker Apex II CCD diffractometer at 296 K using graphite monochromated Mo/Kα radiation (λ = 0.71073 Å). Data reduction was made with the Bruker SAINT program. The structures were solved by direct methods and refined with full-matrix least squares technique using the SHELXTL package.[12] Non-hydrogen atoms were refined with

anisotropic displacement parameters during the final cycles. Organic hydrogen atoms were placed in calculated positions with isotropic displacement parameters set to 1.2 × U_{eq} of the attached atom. The unit cell includes a large region of disordered solvent molecules, which could not be modeled as discrete atomic sites. We employed PLATON/SQUEEZE[13] to calculate the diffraction contribution of the solvent molecules and thereby, to produce a set of solvent-free diffraction intensities; structures were then refined again using the data generated.

A summary of the crystallographic data are given in Table S1. CCDC 1958779, 1958778 contain the supplementary crystallographic data for **NJU-Bai61p** and **NJU-Bai61**. The data can be obtained free of charge at www.ccdc.cam.ac.uk/conts/retrieving.html or from the Cambridge Crystallographic Data Centre, 12, Union Road, Cambridge CB2 1EZ, UK.

Table S1. Crystallographic Data of **NJU-Bai61p** and **NJU-Bai61**

**MOFs**	**NJU-Bai61p**	**NJU-Bai61**
CCDC number	1958779	1958778
Empirical formula	$C_5H_4Cu_2I_2N_2O_2$	$C_{33}H_{45}Cu_{13}I_{11}N_{12}O_7$
Formula weight	505.00	2943.86
Temperature	296 (2) K	173 (2) K
Wavelength	0.71073 Å	0.71073 Å
Crystal system	monoclinic	cubic
Space group	*C* 2/*c*	*I* -43*m*
Unit cell dimensions	*a* = 13.4414(12) Å<br>*b* = 10.3399(9) Å<br>*c* = 15.1736(14) Å<br>*α* = 90 º<br>*β* = 106.964(1) º<br>*γ* = 90 º	*a* = 25.1585(14) Å<br>*b* = 25.1585(14) Å<br>*c* = 25.1585(14) Å<br>*α* = *β* = *γ* = 90 º
Volume [$Å^3$]	2017.1(3)	15924(3)
Z	8	8
Density (calculated)	3.326 g cm^{-3}	2.456 g cm^{-3}
Absorption coefficient	10.306 mm^{-1}	7.699 mm^{-1}
F (000)	1824	10744
Crystal size [mm^3]	0.3 × 0.2 × 0.2	0.25 × 0.25 × 0.25
Theta range for data collection	2.528 to 25.012 º	1.983 to 25.012 º

Limiting indices	-15 < = h < = 15<br>-12 < = k < = 11<br>-18 < = l < = 16	-27 < = h < = 29<br>-29 < = k < = 29<br>-29 < = l < = 29
Reflections collected unique<br>Reflections unique	7232 / 1575<br><br>[R(int) = 0.0248]	59047 / 2593<br><br>[R(int) = 0.1345]
Completeness	98.9%	99.9%
Data/restraints/parameters	1757 / 0 / 120	2593 / 12 / 137
Goodness-of-fit on F^2	1.237	1.184
Final R indices [I>2sigma(I)]	R1 = 0.0378, wR2[a] = 0.0983	R1 = 0.0649, wR2[a] = 0.1819
R indices (all data)	R1 = 0.0385, wR2[a] = 0.0985	R1 = 0.0766, wR2[a] = 0.1992
Largest diff. peak and hole	3.178 and -1.096 e. $Å^{-3}$	3.449 and -3.014 e. $Å^{-3}$

[a] $R1 = \Sigma||F_o| - |F_c||/|F_o|$; $wR2 = [\Sigma w(\Sigma F_o^2 - F_c^2)^2/\Sigma w(F_o^2)^2]^{1/2}$.

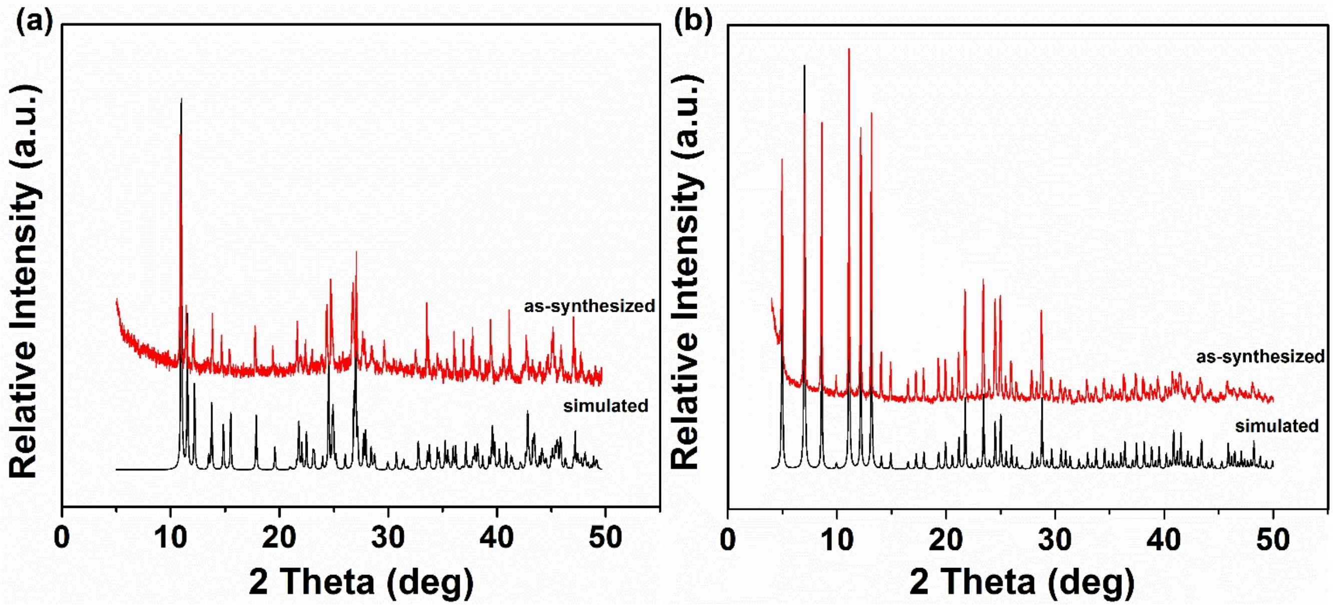

Figure S1. The PXRD patterns of **NJU-Bai61p** (a) and **NJU-Bai61** (b).

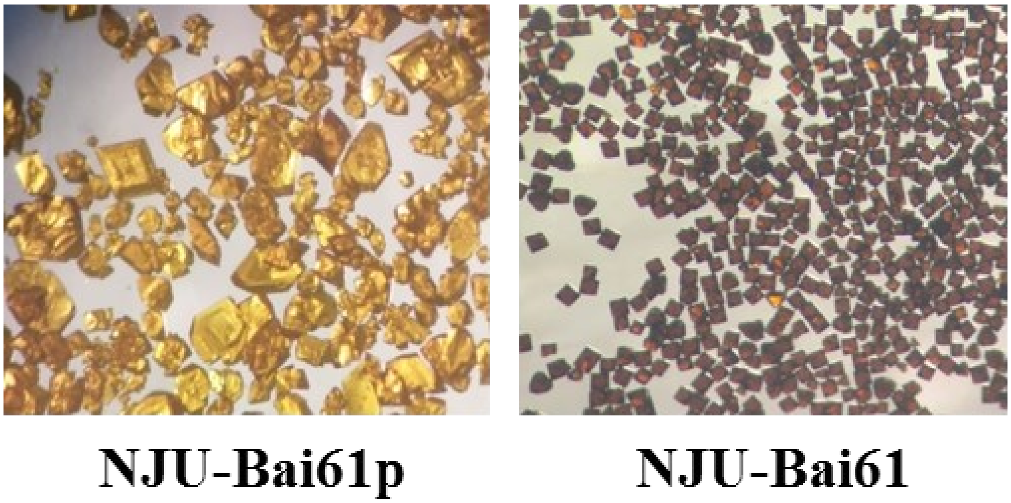

Figure S2. Optical microscope images of crystals of **NJU-Bai61p** and **NJU-Bai61**.

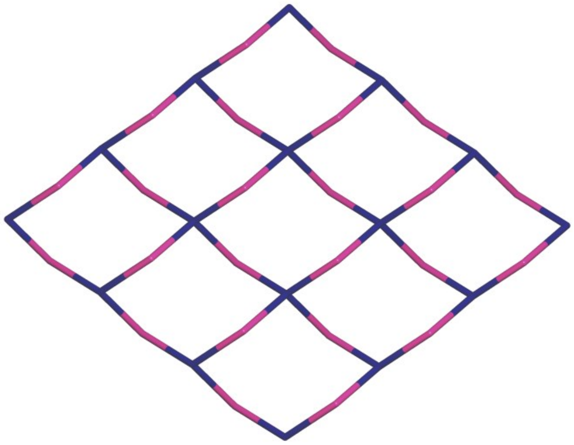

Figure S3. The sql topology of **NJU-Bai61p**.

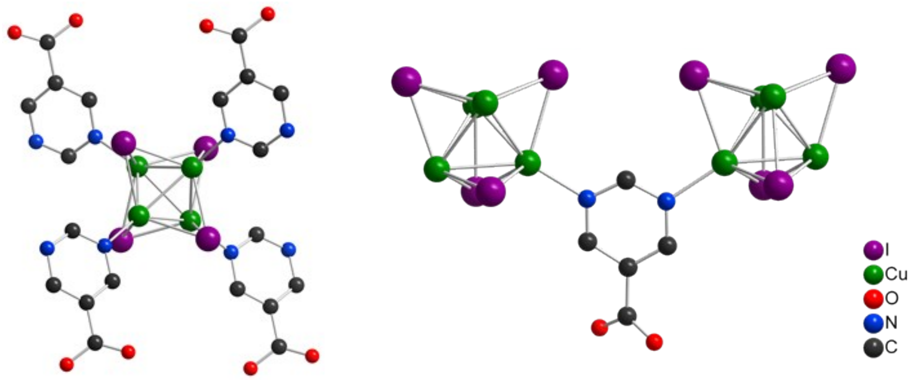

Figure S4. Coordination environments of Cu_4I_4 cluster and Hpmc ligand in **NJU-Bai61p**, respectively.

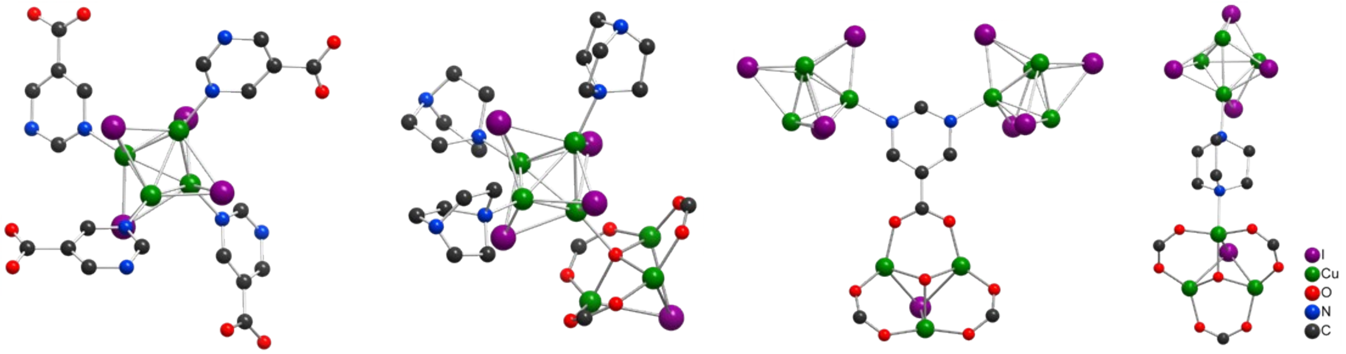

Figure S5. Coordination environments of Cu_4I_4 cluster, Hpmc and Dabco ligands in **NJU-Bai61**, respectively.

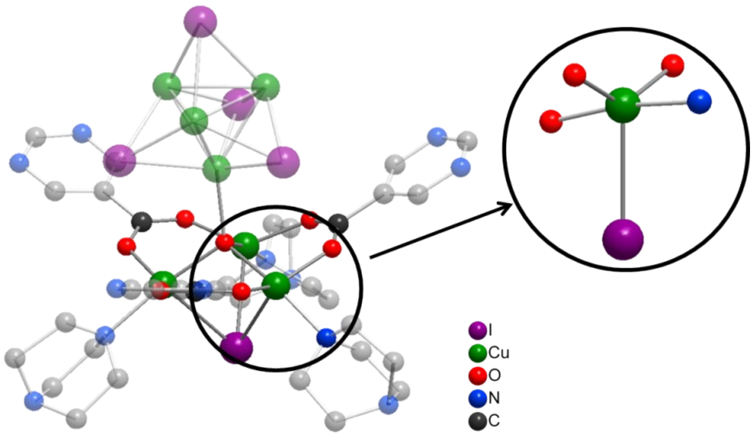

Figure S6. Coordination environment of $Cu_3OI(CO_2)_3$ cluster and coordination geometry around Cu(II) ion in **NJU-Bai61**.

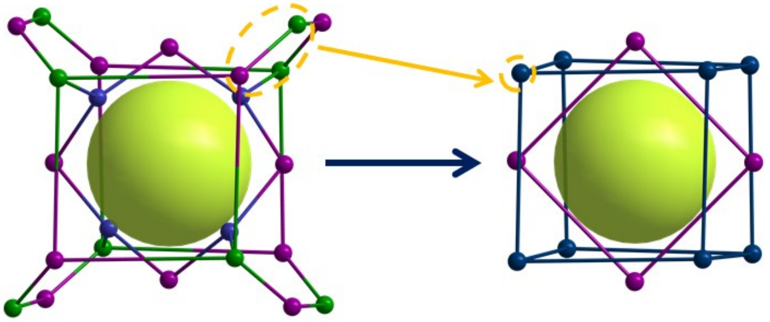

Figure S7. The simplified representation of the cage B in **NJU-Bai61**. Cu_4I_4 clusters, violet ellipsoids; $Cu_3OI(CO_2)_3$ clusters, green ellipsoids; Hpmc ligand, indigo ellipsoids; [Cu_4I_4-$Cu_3OI(CO_2)_3$] linkage clusters, dark teal ellipsoids.

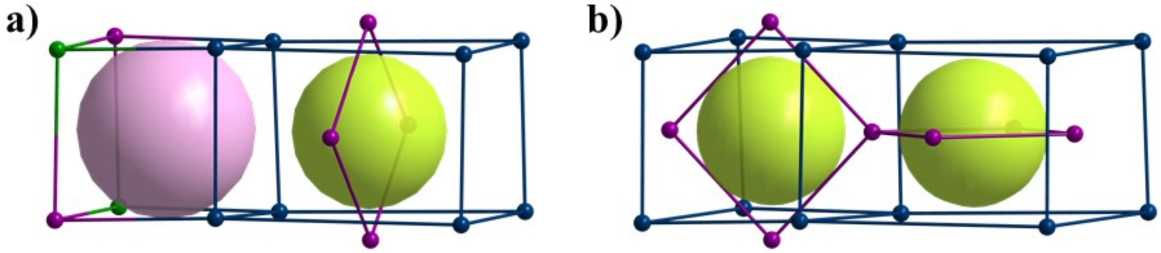

Figure S8. a) The cages A and B connect with each other through sharing the quadrilateral window; b) the cages B connect with each other sharing the facet including a quadrilateral window and a Cu_4I_4 cluster.

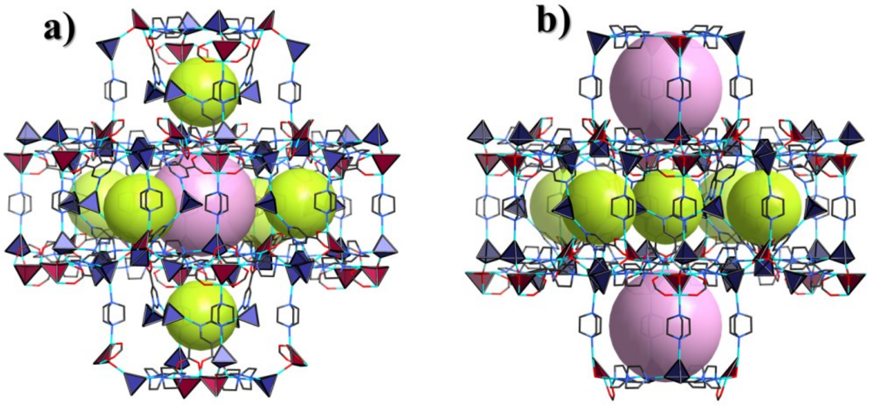

Figure S9. a) Every cage A shares facets with six cages B; b) Every cage B shares facets with two cages A and four cages B.

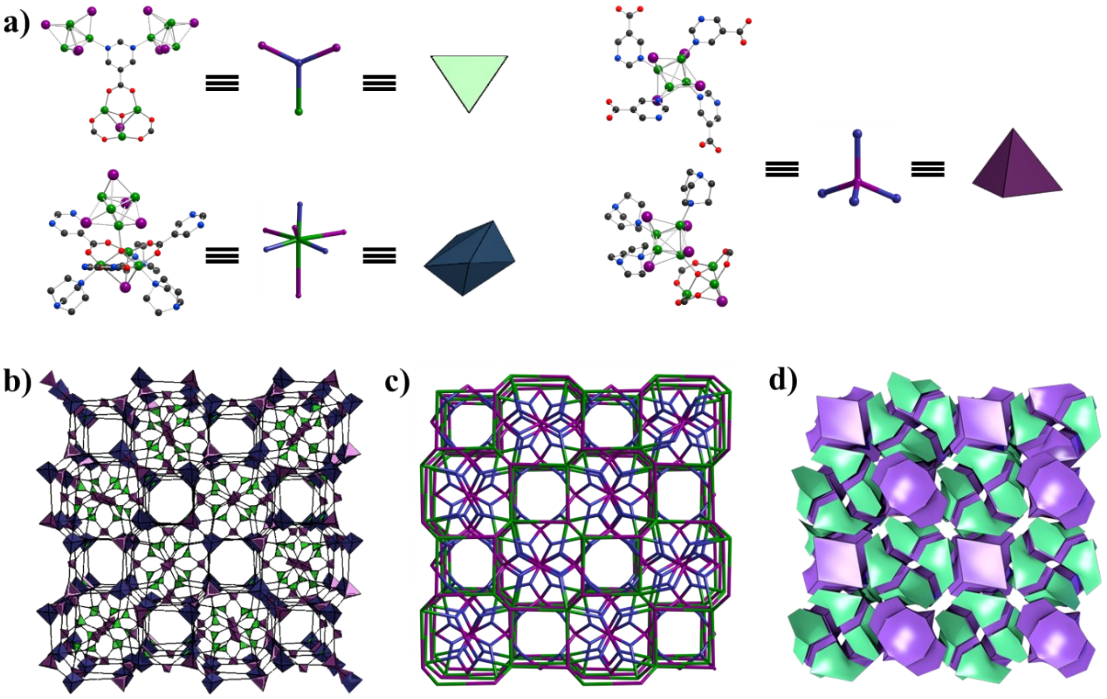

Figure S10. Description of **NJU-Bai61**: (a) The coordination environments of pmc ligand viewed as a 3-connected triangular node, the Cu_4I_4 cluster viewed as a 4-connected tetrahedral node, and the $Cu_3OI(CO_2)_3$ cluster viewed as a 7-connected single cap octahedron node; (b) polyhedral view of the topology; (c) the (3,4,4,7)-connected net new topology; (d) the tiling illustration.

Section 4. IR and XPS analyses

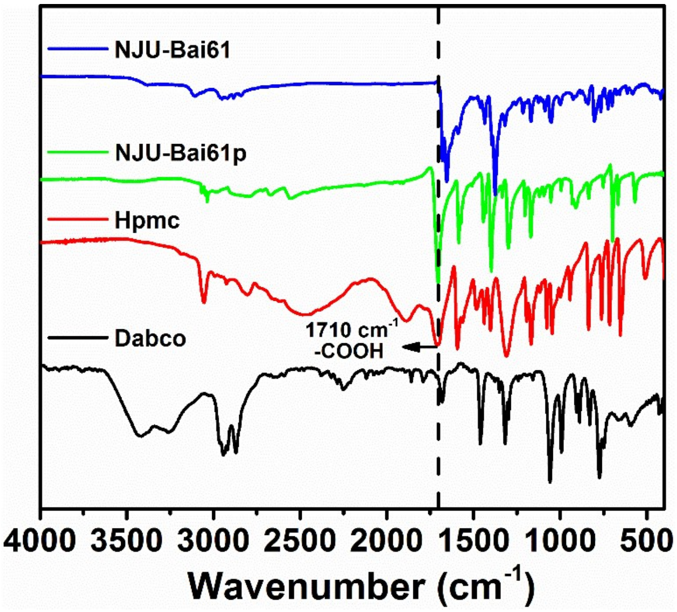

Figure S11. IR spectra of **NJU-Bai61p** and **NJU-Bai61**.

The peak at 1710 cm^{-1} could be assigned to the characteristic peak of -COOH in Hpmc ligand and **NJU-Bai61p**. This peak was not observed in **NJU-Bai61** indicating that all the -COOH groups in **NJU-Bai61** were participated in the coordination with Cu(II) ions.

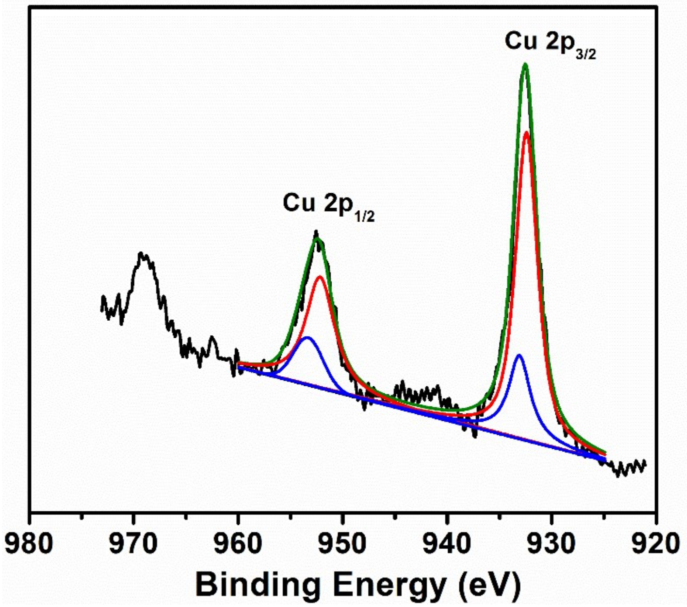

Figure S12. XPS spectra of **NJU-Bai61**. (red and blue lines are the peak of Cu(I) and Cu(II), respectively.)

The characteristic peaks of $2p_{3/2}$ and $2p_{1/2}$ at 932.4 and 952.2 eV indicate the presence of Cu(I) and the remaining peaks at 933.1 and 953.3 eV correspond to the Cu(II) in **NJU-Bai61**. Analysis of the area ratio of Cu(I) and Cu(II) was 77 : 23, in according with the calculated balance of charge of the formula and crystallographic findings of 10 Cu(I) : 3Cu(II).[14]

Section 5. Stability tests

5.1 Thermal stability tests

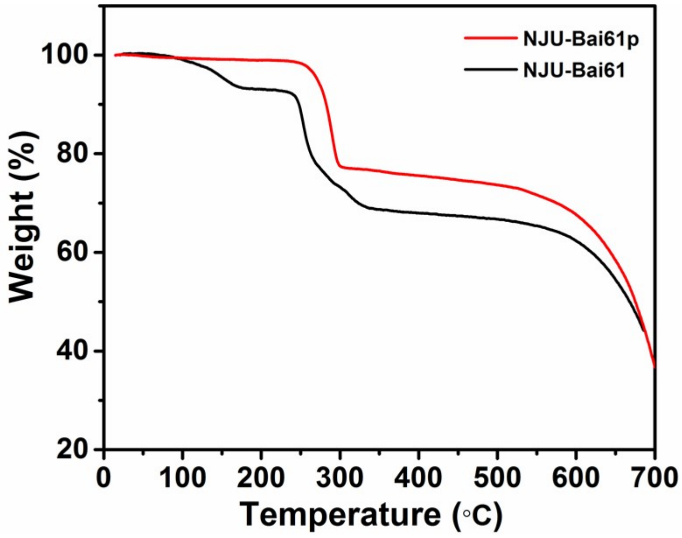

Figure S13. TG curves of **NJU-Bai61p** (red) and **NJU-Bai61** (blank).

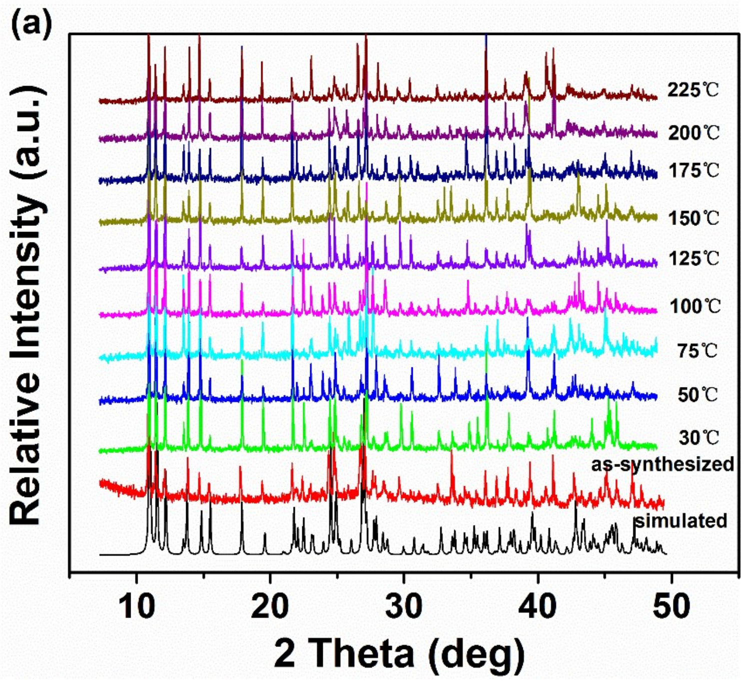

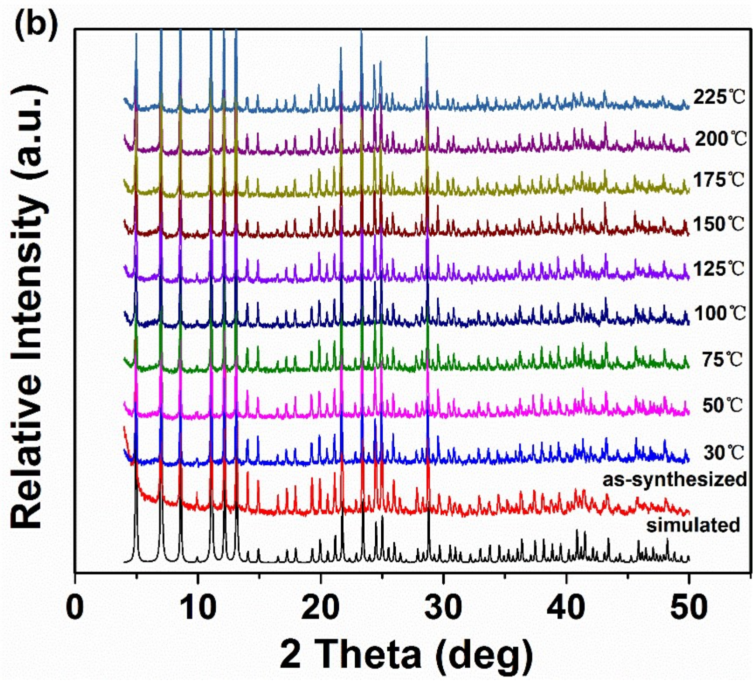

Figure S14. VT-PXRD patterns of **NJU-Bai61p** and **NJU-Bai61** under vacuum: (a) for **NJU-Bai61p**; (b) for **NJU-Bai61**.

5.2 Chemical stability tests

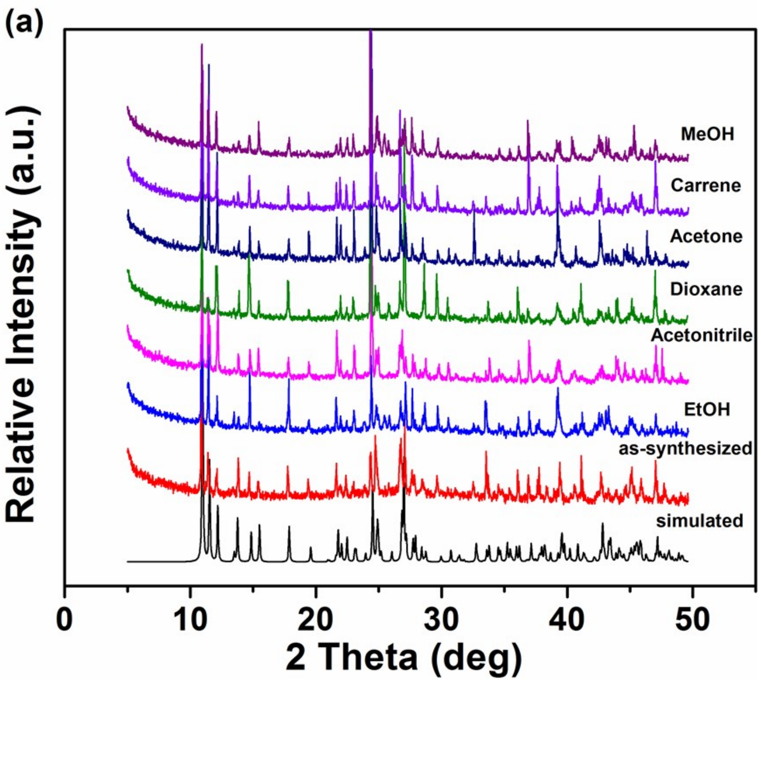

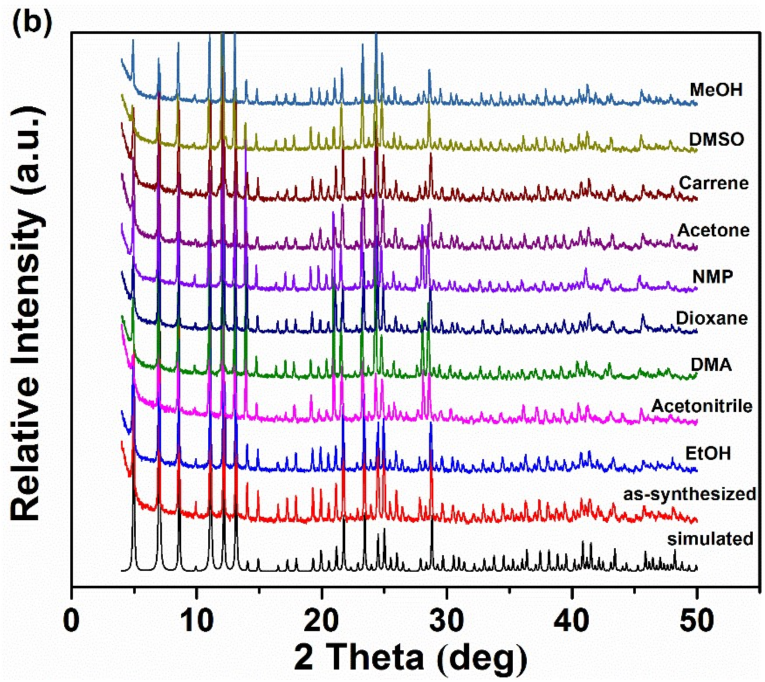

Figure S15. PXRD patterns of **NJU-Bai61p** and **NJU-Bai61** after immersing in different solvents for 24 hours: (a) for **NJU-Bai61p**; (b) for **NJU-Bai61**.

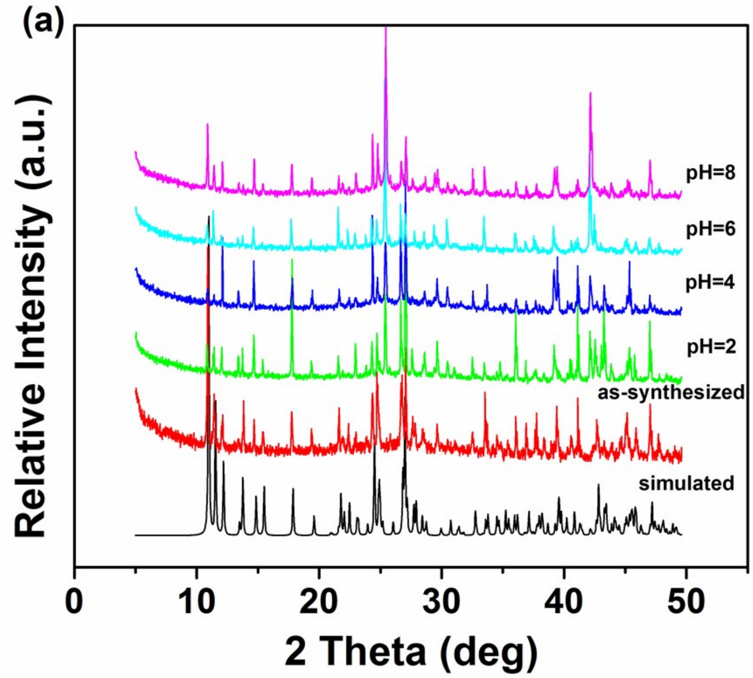

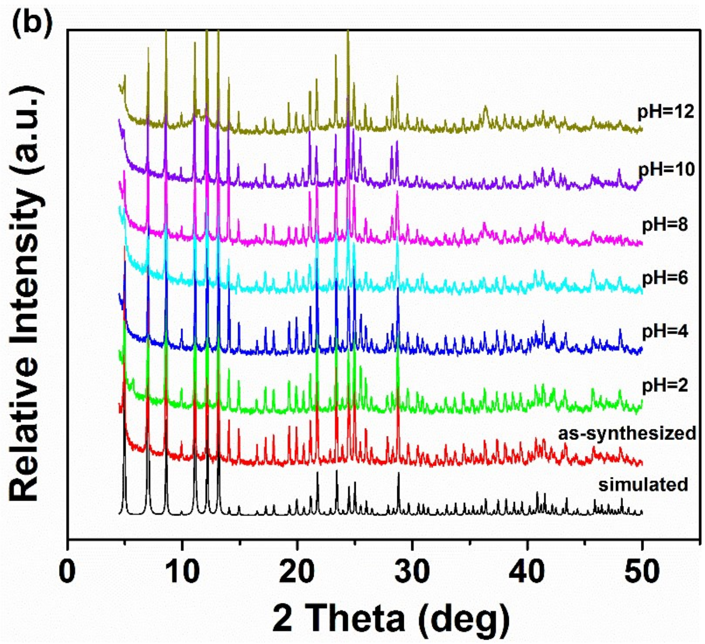

Figure S16. PXRD patterns of **NJU-Bai61p** and **NJU-Bai61** after immersing in HCl/KOH aqueous solutions of different pH for 24 hours: (a) for **NJU-Bai61p**; (b) for **NJU-Bai61**.

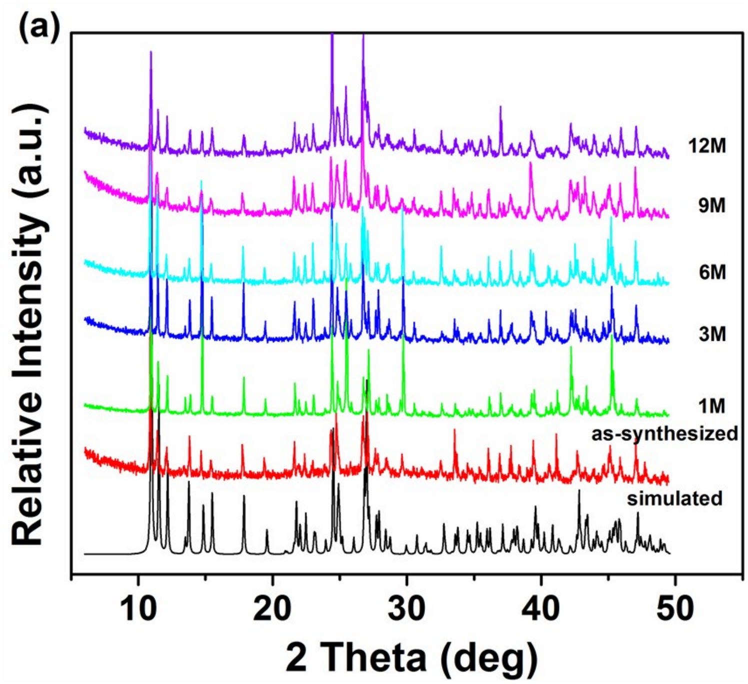

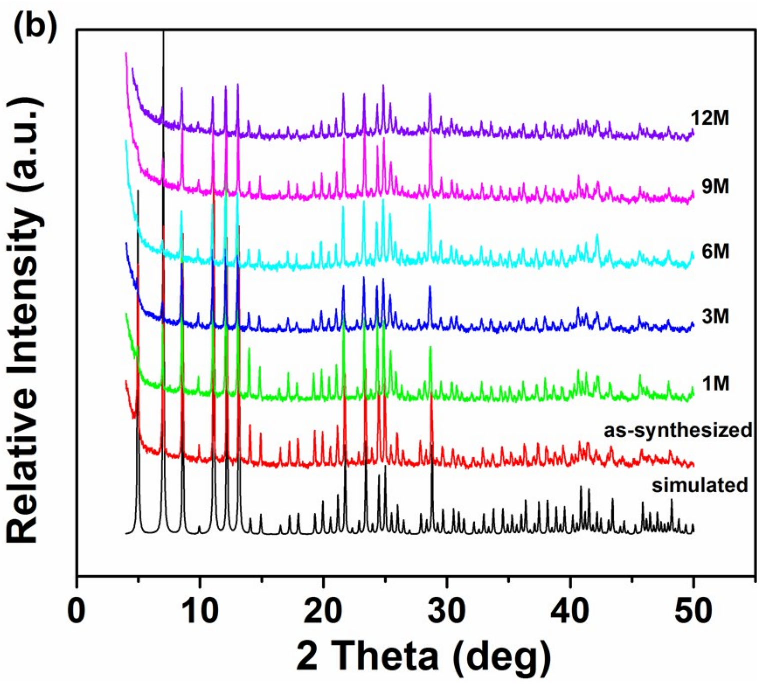

Figure S17. PXRD patterns of **NJU-Bai61p** and **NJU-Bai61** immersing in water for different time: (a) for **NJU-Bai61p**; (b) for **NJU-Bai61**.

Section 6. Characterization of band structure

Table S2. Main calculated optical transitions for **NJU-Bai61p**.

Calcd. (nm)	*f*	Optical transition	Character
400.15	0.0053	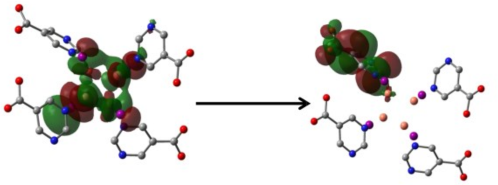<br>HOMO-3 LUMO	Cu_4I_4 cluster to linker charge transfer
419.55	0.0028	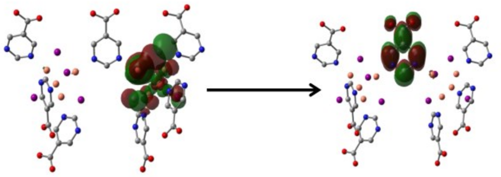<br>HOMO-8 LUMO	Cu_4I_4 cluster to linker charge transfer
472.72	0.0009	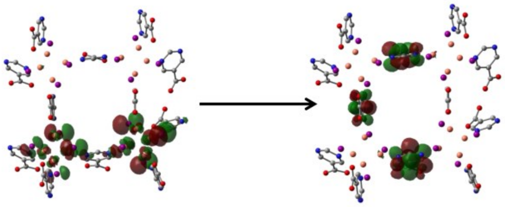<br>HOMO-2 LUMO+4	Cu_4I_4 cluster to linker charge transfer
477.18	0.0012	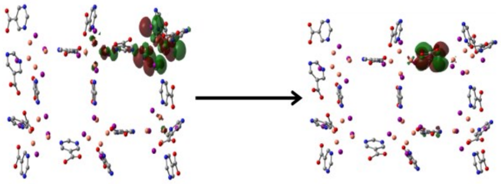<br>HOMO-2 LUMO+12<br>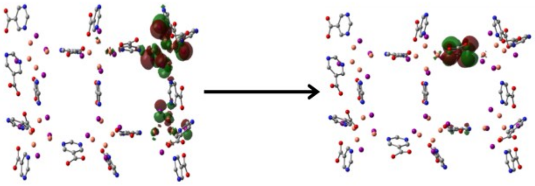<br>HOMO-1 LUMO+12	Cu_4I_4 cluster to linker charge transfer
578.37	0.0003	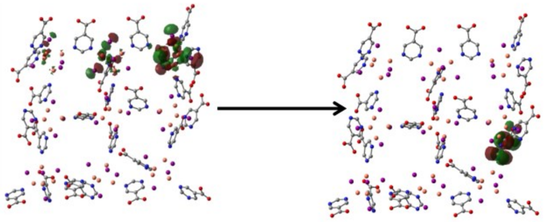<br>HOMO-9 LUMO	Cu_4I_4 cluster to linker charge transfer

Table S3. Main calculated optical transitions for **NJU-Bai61**.

Calcd. (nm)	*f*	Optical transition	Character
393.19	0.0058	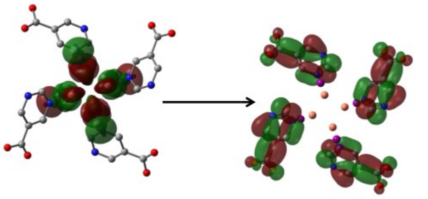<br>HOMO-3 LUMO+1	Cu_4I_4 cluster to linker charge transfer
623.17	0.0833	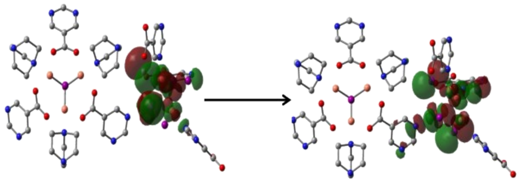<br>366β 372β	Intra-Cu_4I_4 cluster charge transfer
690.22	0.0587	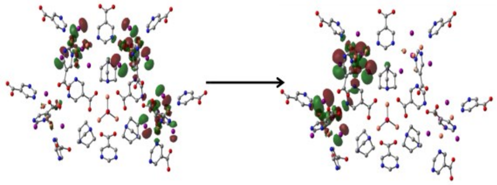<br>771β 784β	Cu_4I_4 cluster to Cu_4I_4 cluster charge transfer
815.91	0.0673	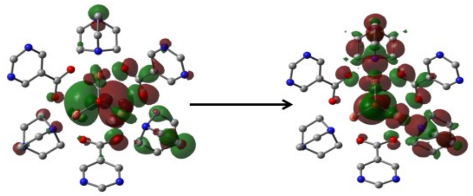<br>218β 224β	$Cu_3OI(CO_2)_3$ cluster to linker charge transfer
940.58	0.0209	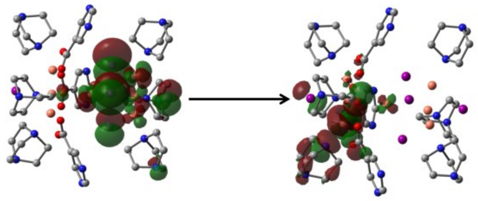<br>365β 370β	Cu_4I_4 cluster to $Cu_3OI(CO_2)_3$ cluster charge transfer
958.93	0.0205	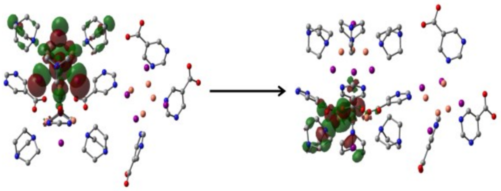<br>513β 518β	Cu_4I_4 cluster to $Cu_3OI(CO_2)_3$ cluster charge transfer

1065.95	0.0226	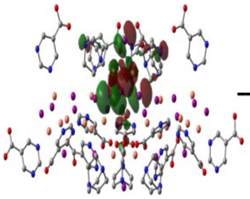 894β	→	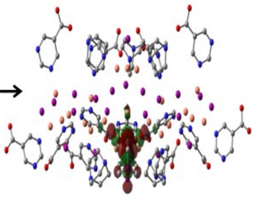 898β	Cu_4I_4 cluster to $Cu_3OI(CO_2)_3$ cluster charge transfer
1130.89	0.0525	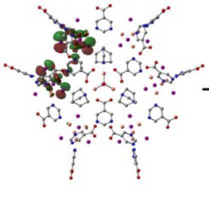 1014β	→	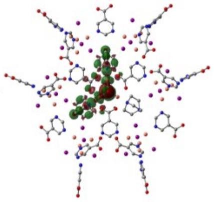 1016β	Cu_4I_4 cluster to $Cu_3OI(CO_2)_3$ cluster charge transfer

Table S4. The calculated HOMO-LUMO gaps and 3D plots of the HOMO and LUMO of several clusters in the **NJU-Bai61p**.

Compound	HOMO	LUMO	ΔE_{gap}
Cluster 1	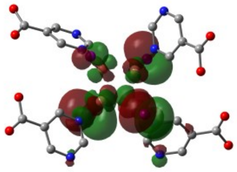<br>-5.83 eV	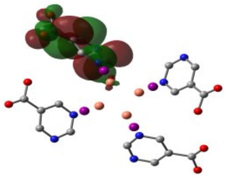<br>-2.46 eV	3.37 eV
Cluster 2	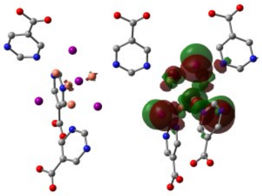<br>-5.73 eV	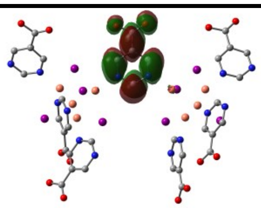<br>-2.50 eV	3.23 eV
Cluster 3	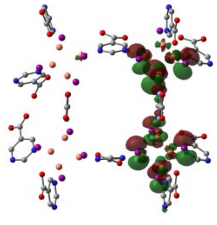<br>-5.92 eV	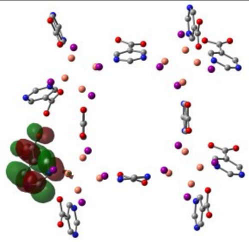<br>-3.25 eV	2.67 eV
Cluster 4	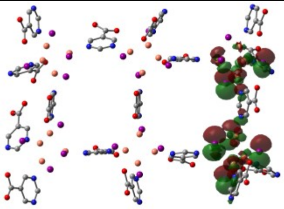<br>-5.87 eV	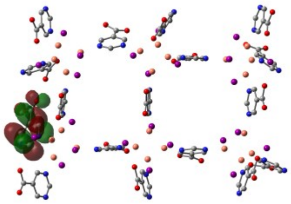<br>-3.27 eV	2.60 eV
Cluster 5	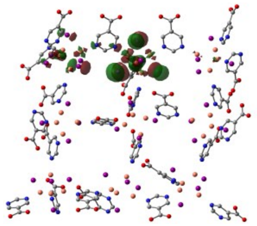<br>-5.61 eV	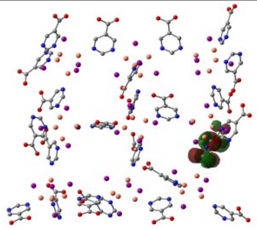<br>-3.45 eV	2.16 eV

$\Delta E_{gap} = E_{LUMO}-E_{HOMO}$

Table S5. The calculated HOMO-LUMO gaps and 3D plots of the HOMO and LUMO of several clusters in the **NJU-Bai61**.

Compound	HOMO	LUMO	ΔE_{gap}
Cluster 1	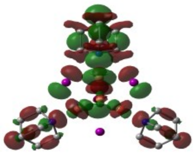 -5.48 eV	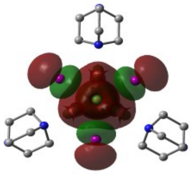 -1.24 eV	4.24 eV
Cluster 2	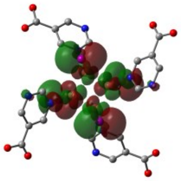 -5.70 eV	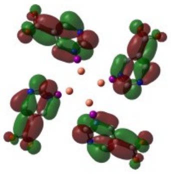 -2.27 eV	3.43 eV
Cluster 3	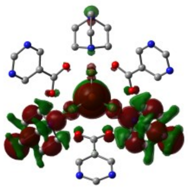 -5.88 eV	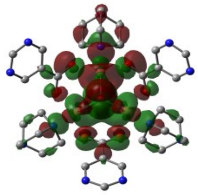 -3.31 eV	2.57 eV
Cluster 4	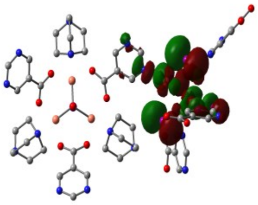 -5.75 eV	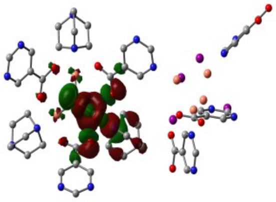 -3.26 eV	2.49 eV
Cluster 5	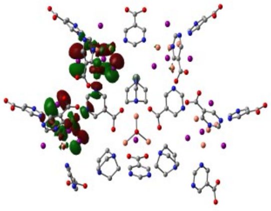 -5.83 eV	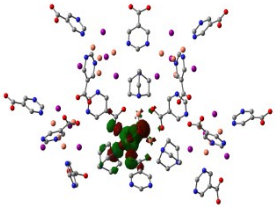 -3.50 eV	2.33 eV
Cluster 6	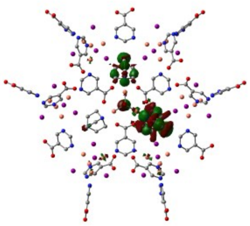	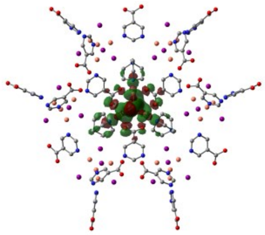	2.42 eV

	-5.76 eV	-3.34 eV	
Cluster 7	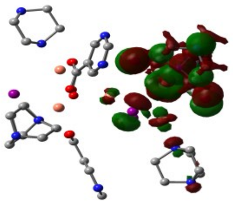 -5.02 eV	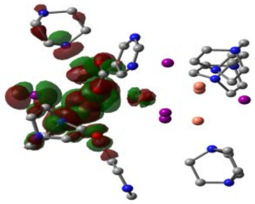 -3.46 eV	1.56 eV
Cluster 8	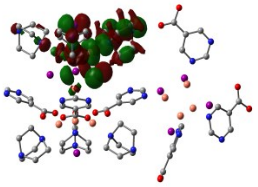 -4.95 eV	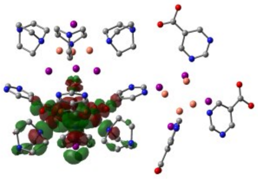 -3.43 eV	1.52 eV
Cluster 9	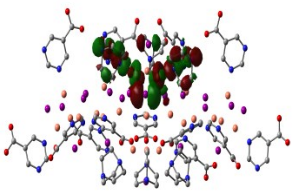 -4.86 eV	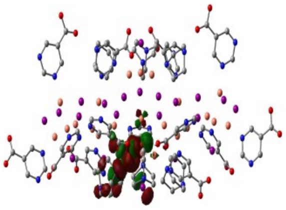 -3.61 eV	1.25 eV

$\Delta E_{gap} = E_{LUMO} - E_{HOMO}$

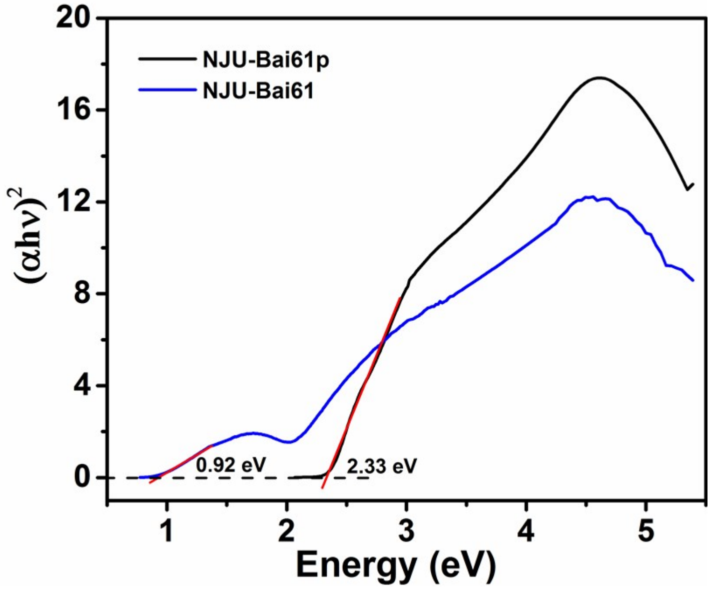

Figure S18. Tauc plots of **NJU-Bai61p** and **NJU-Bai61**.

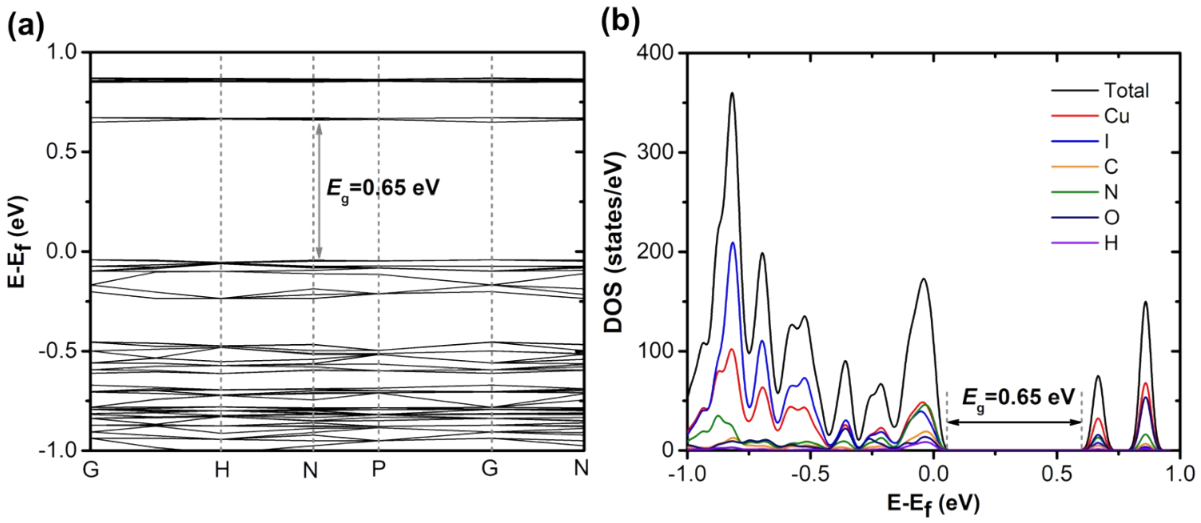

Figure S19. (a) The band structure and (b) the density of states (DOS) of **NJU-Bai61**.

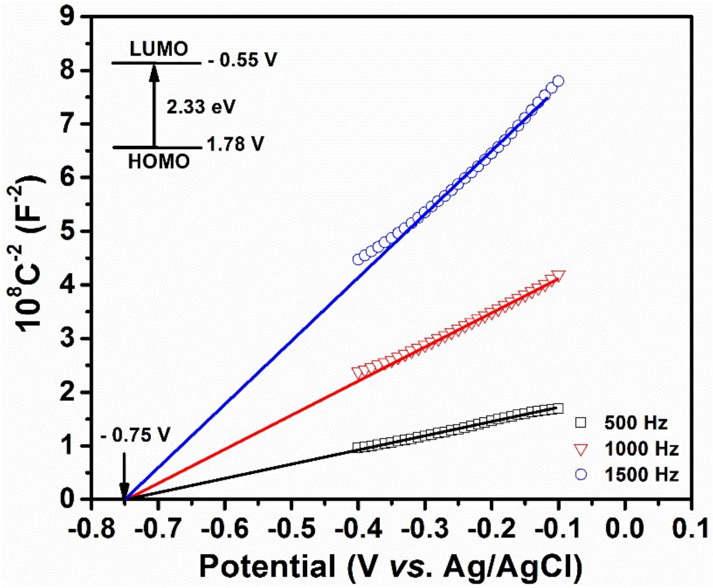

Figure S20. Mott-Schottky plots for **NJU-Bai61p**.

Section 7. Electrical conductivity tests

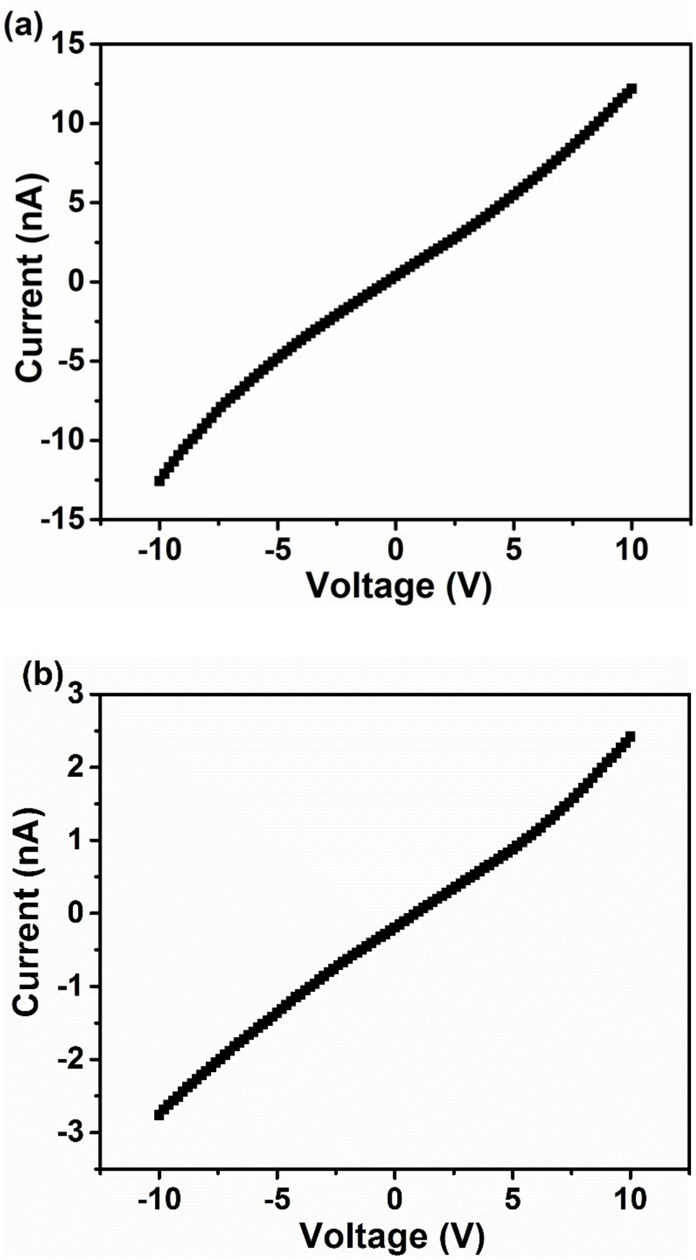

Figure S21. I-V curves of **NJU-Bai61p** and **NJU-Bai61**: (a) for **NJU-Bai61p**; (b) for **NJU-Bai61**.

Table S6. The parameters of **NJU-Bai61p** and **NJU-Bai61** used for calculating of electrical conductivity.

	L (cm)	A (cm^2)	G (S)	σ (S/cm)
**NJU-Bai61p**	0.094	1.28 e-3	1.13 e-9	8.30 e-8
**NJU-Bai61**	0.074	1.37 e-3	2.41 e-10	1.30 e-8

Section 8. The research of photocatalytic CO_2 reduction reaction

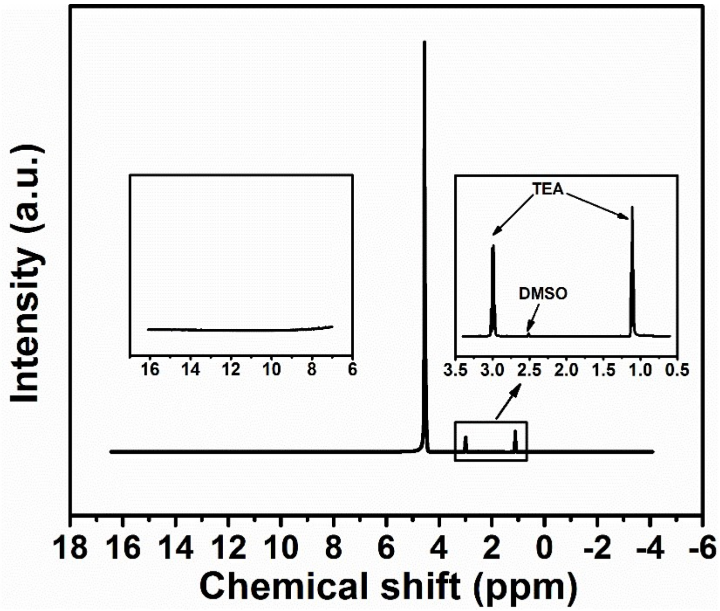

Figure S22. ^{1}H-NMR spectrum of the filtrate of the photocatalytic CO_2 reduction using **NJU-Bai61** as the catalyst.

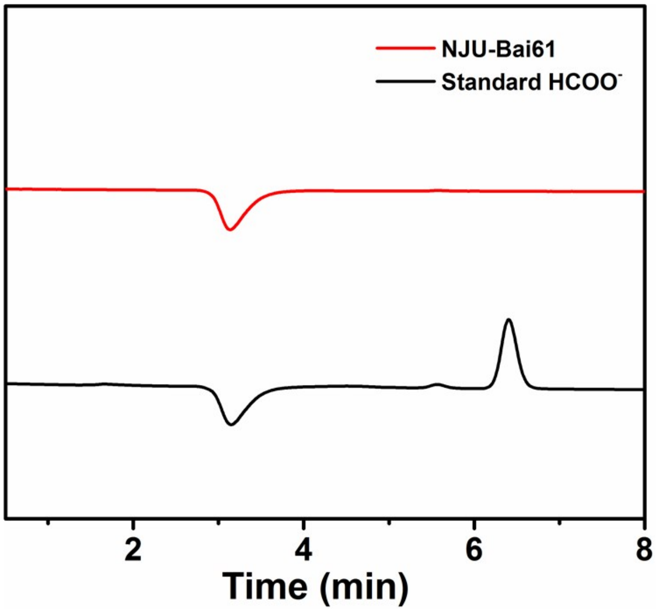

Figure S23. Ion chromatograph of the filtrate of the photocatalytic CO_2 reduction using **NJU-Bai61** as the catalyst.

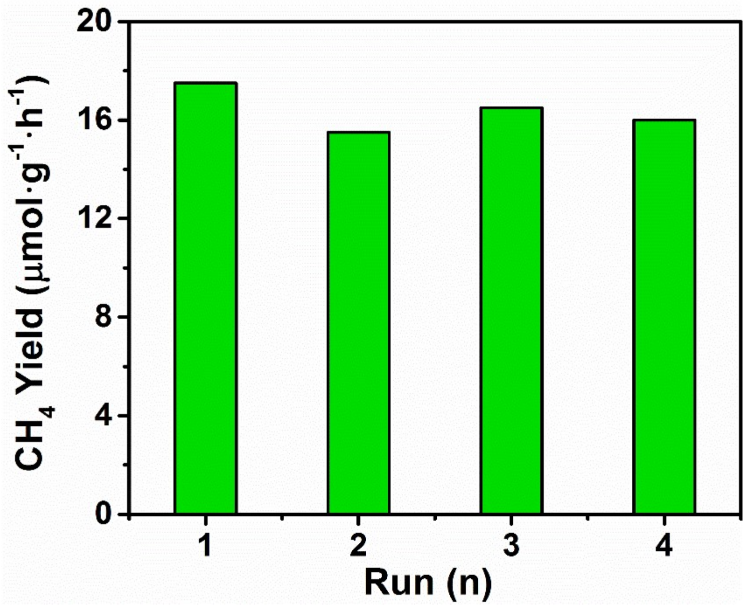

Figure S24. The yield of CH_4 produced in four repeated photocatalytic reactions using **NJU-Bai61** as catalysts.

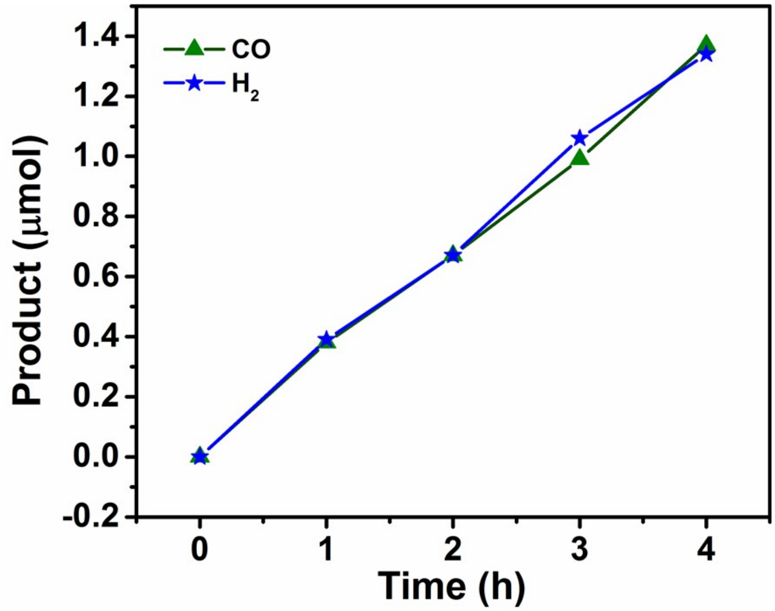

Figure S25. The amounts of CO and H_2 produced as a function of the irradiation time over **NJU-Bai61p**.

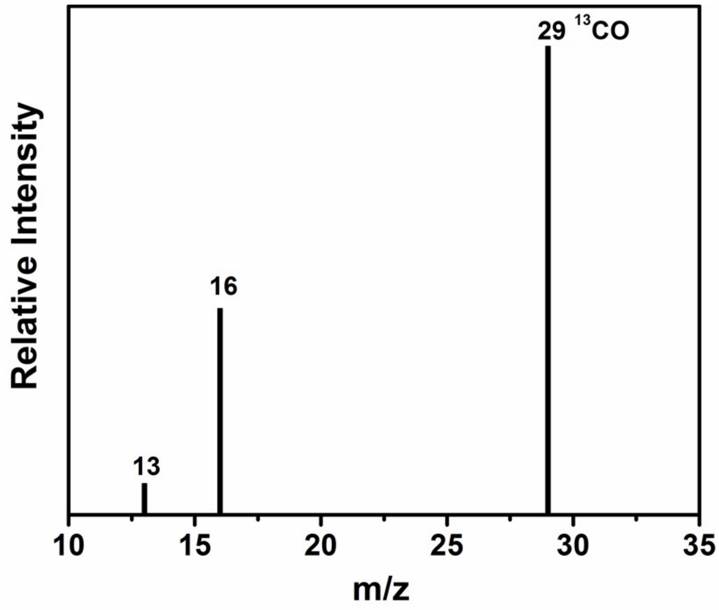

Figure S26. The mass spectra analysis of ^{13}CO recorded under a $^{13}CO_2$ atmosphere using **NJU-Bai61p** as the catalyst.

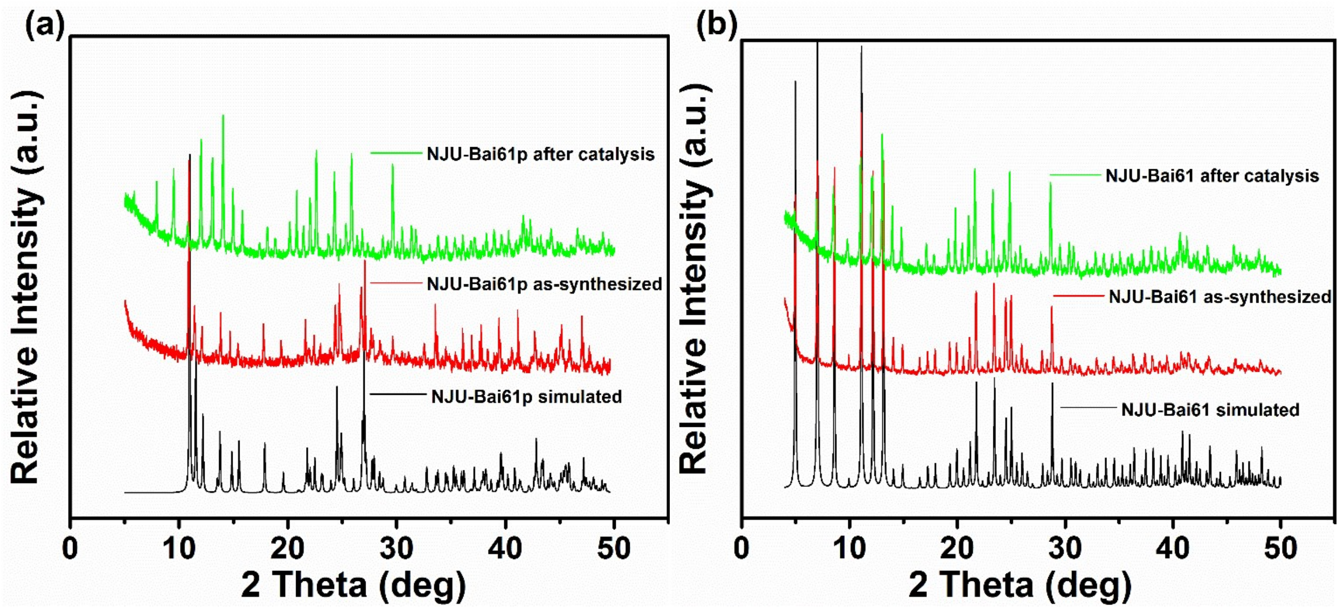

Figure S27. The XRD patterns of **NJU-Bai61p** and **NJU-Bai61** after the photocatalytic experiments: (a) for **NJU-Bai61p**; (b) for **NJU-Bai61**.

Table S7. Control photocatalytic experiments using **NJU-Bai61** as catalyst.

Entry	CH_4 (μmol)	CO (μmol)	H_2 (μmol)	CH_4 - TON[a]	CH_4 - TOF[b] (h^{-1})	CH_4 Selectivity
1[c]	n.d.[d]	n.d.	n.d.	-	-	-
2[e]	n.d.	n.d.	n.d.	-	-	-
3[f]	n.d.	n.d.	n.d.	-	-	-
4[g]	n.d.	n.d.	n.d.	-	-	-
5[h]	n.d.	n.d.	n.d.	-	-	-
6[i]	n.d.	trace	n.d.	-	-	-
**7[j]**	**1.26**	**0.32**	**0.15**	**0.063**	**0.016**	**72.8%**

Reaction conditions: [a] Turnover number = $n_{CH4}/n_{Cu^{2+}}$, where n_{CH4} is the amount of CH_4 (mol), $n_{Cu^{2+}}$ is the amount of Cu^{2+} of the catalyst (mol). [b] Turnover frequency = TON/t, where t is the reaction time (h). [c] Without **NJU-Bai61**. [d] Not detectable. [e] In the dark. [f] Without TEA. [g] using Ar to replace pure CO_2. [h] Using ligand (Hpmc or Dabco) to replace catalyst. [i] Using Ligands (Hpmc and Dabco), CuI and $CuCl_2$ mixture to replace catalyst. [j] Standard reaction condition: H_2O/TEA = 45mL/5mL, 20 mg catalyst, $300 < \lambda < 2500$ nm, 25 °C, 4 h.

There was no CH_4 detected in the absence of catalyst, illumination, TEA, using ligands or the ligands, CuI and $CuCl_2$ mixture directly, which indicates that **NJU-Bai61**, light and sacrificial agent maintain the photocatalytic progress together. When Ar was used instead of CO_2, CH_4 was not been detected, indicating that CH_4 was reduced from CO_2 rather than skeleton decomposition.

Table S8. Summarry of the photocatalytic selectivity and activity of reported MOFs and MOF-based composites for converting CO_2 to CH_4.

MOFs	The role of MOFs	Light (nm)	Reaction agent	product formation rate ($\mu mol\ g^{-1}\ h^{-1}$)	CH_4 selectivity	Res.
**NJU-Bai61**	**photocatalyst**	**300 < λ < 2500**	**H_2O - TEA (9:1 *v/v*)**	**CH_4: 15.75 CO: 4.0 H_2: 1.87**	**72.8%**	
MOF-525-Co	photocatalyst	400 < λ < 800	MeCN - TEOA (4:1 *v/v*)	CH_4: 36.76 CO: 200.6	15.5%	[15]
ZrPP-1-Co	photocatalyst	λ > 420	MeCN - TEOA (4:1 *v/v*)	CH_4: 0.5 CO: 14	3.4%	[16]
BIF-20@g-C_3N_4 (20 wt%)	cocatalyst	400 < λ < 800	MeCN - TEOA (4:1 *v/v*)	CH_4: 15.52 CO: 53.87	22.4%	[17]
NH_2-UiO-66/$CsPbBr_3$	cocatalyst	λ > 420	EtOAc - H_2O (300:1 *v/v*)	CH_4: 0.26 CO: 8.21	3.1%	[18]
$MAPbI_3$@PCN-221($Fe_{0.2}$)	cocatalyst	λ > 400	MeCN - H_2O (1:0.012 *v/v*)	CH_4: 12.87 CO: 6.63	66.0%	[19]
$CsPbBr_3$/ZIF-8(0.5,2)	cocatalyst	λ > 420	CO_2 and H_2O vapor	CH_4: 1.58 CO: 0.52	75.2%	[20]
TiO_2/Co-ZIF-9	cocatalyst	200 < λ < 900	CO_2 and H_2O vapor	CH_4: 2.0 CO: 17.6 H_2: 2.6	9.0%	[21]
TiO_2/$Cu_3(BTC)_2$	cocatalyst	λ < 400	CO_2 and H_2O vapor	CH_4: 2.64	100%	[22]
TiO_2/Cu_2O/$Cu_3(BTC)_2$	cocatalyst	λ > 320	CO_2 and H_2O vapor	CH_4: 155 CO: 85	64.6%	[23]
TiO_2/Mg-CPO-27	cocatalyst	λ = 365	CO_2 and H_2O vapor	CH_4: 2.35 CO: 4.09 O_2: 6.29	18.4%	[24]
MIL-101(Cr)-Ag	cocatalyst	400 < λ < 780	CO_2 TEOA (2 mL)	CH_4: 427.5 CO: 808.2 H_2: 82.1	32.4%	[25]

Section 9. The research of photocatalytic pathway of CO_2-to-CH_4 reduction

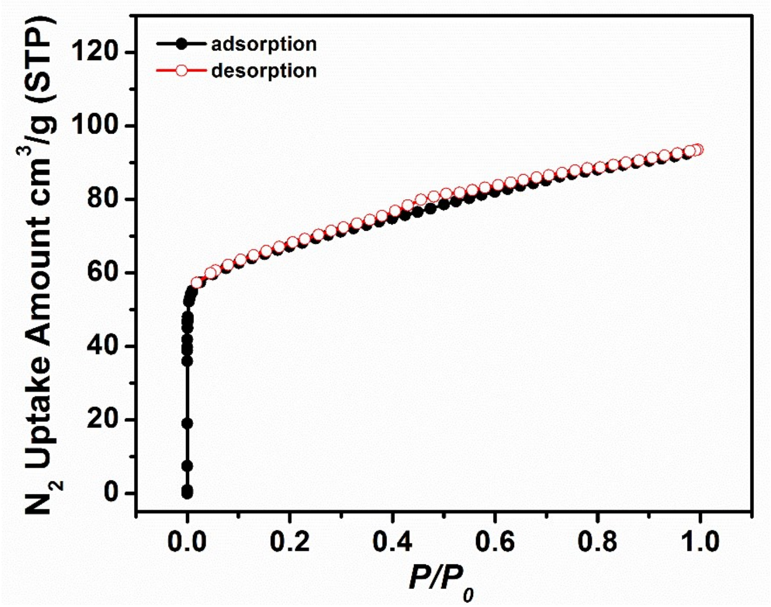

Figure S28. N_2 adsorption and desorption isotherms of **NJU-Bai61** at 77 K.

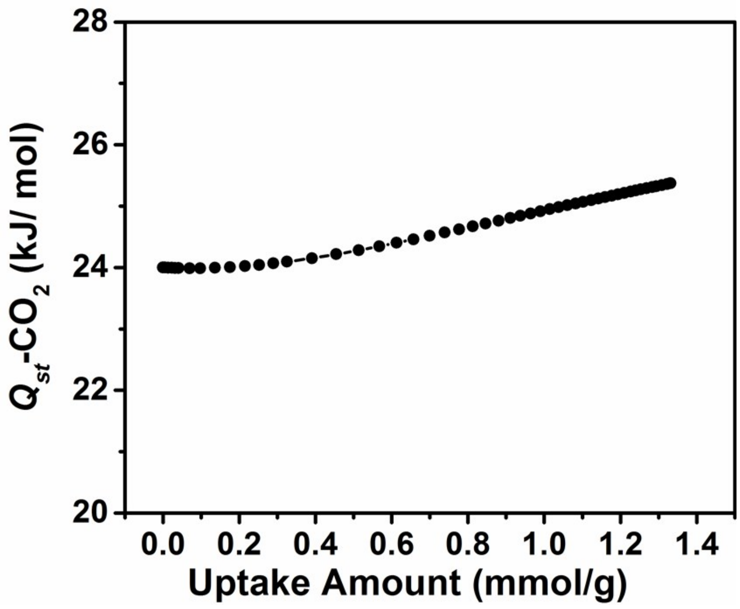

Figure S29. The isosteric CO_2 adsorption enthalpy of **NJU-Bai61**.

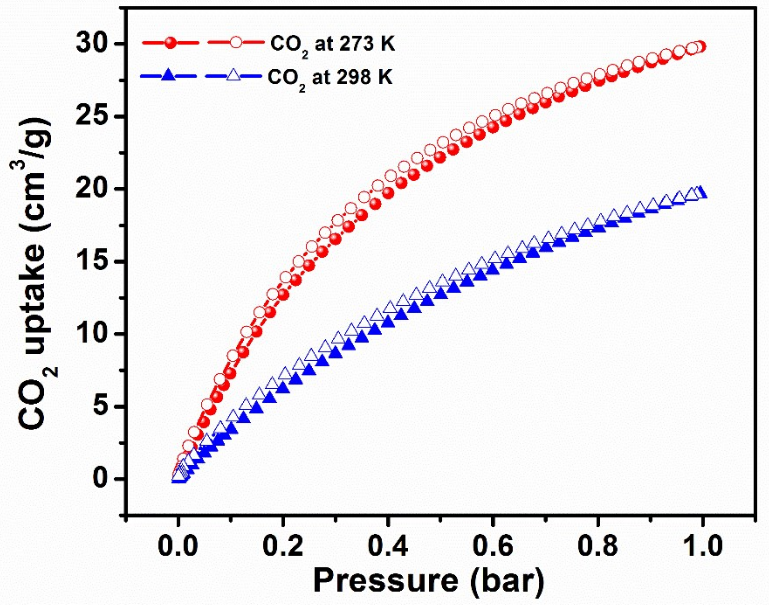

Figure S30. CO_2 adsorption isotherms of the **NJU-Bai61.**

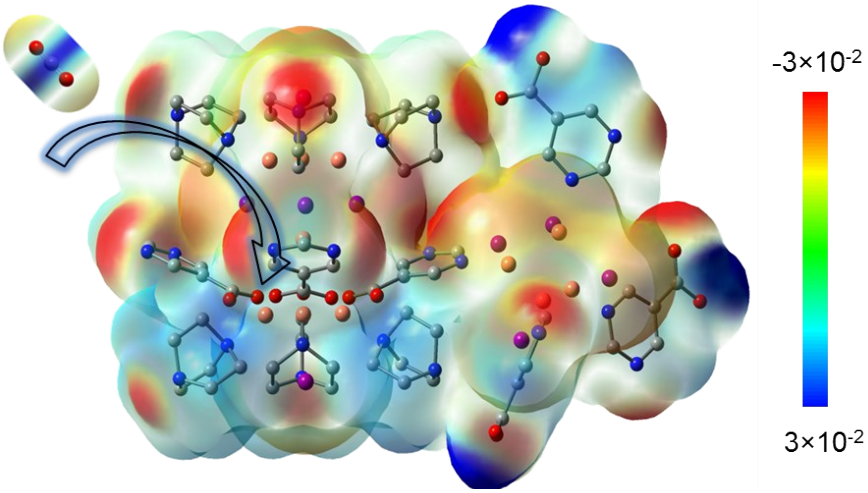

Figure S31. The electrostatic potential map of **NJU-Bai61** (isovalue = 0.0004).

The electrostatic potential mapped surface of **NJU-Bai61** was calculated by the B3LYP/6-31G(d) (isovalue = 0.0004). The different values of the electrostatic potential at the surface were represented by different color codes in the ranges between -0.03 a.u. (deepest red) to 0.03 a.u. (deepest blue). The Mulliken charges of the oxygen atoms and carbon atom are negative represented by yellow and positive represented by blue in CO_2, respectively. Mulliken charges of Cu(II) atoms of $Cu_3OI(CO_2)_3$ cluster are 0.435, 0.429 and 0.416 a.u. Mulliken charges of Cu(I) atoms of Cu_4I_4 clusters are -0.07, -0.07, 0.002, -0.065, -0.171, -0.151, -0.172 and 0.242 a.u.

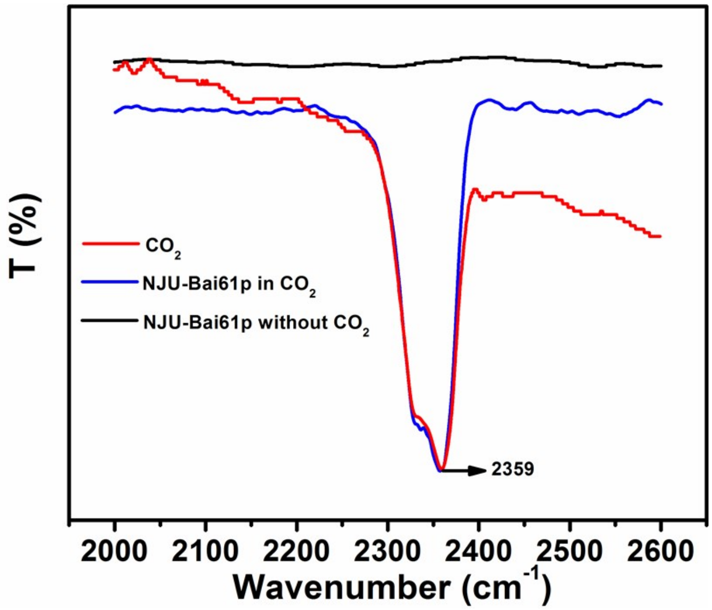

Figure S32. In site FTIR spectra of CO_2 and **NJU-Bai61p** in and without CO_2 atmosphere.

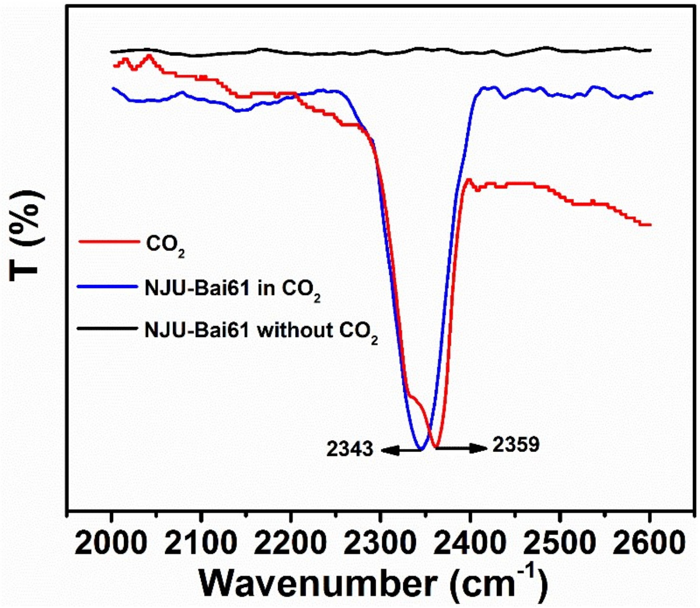

Figure S33. In site FTIR spectra of CO_2 and **NJU-Bai61** in and without CO_2 atmosphere.

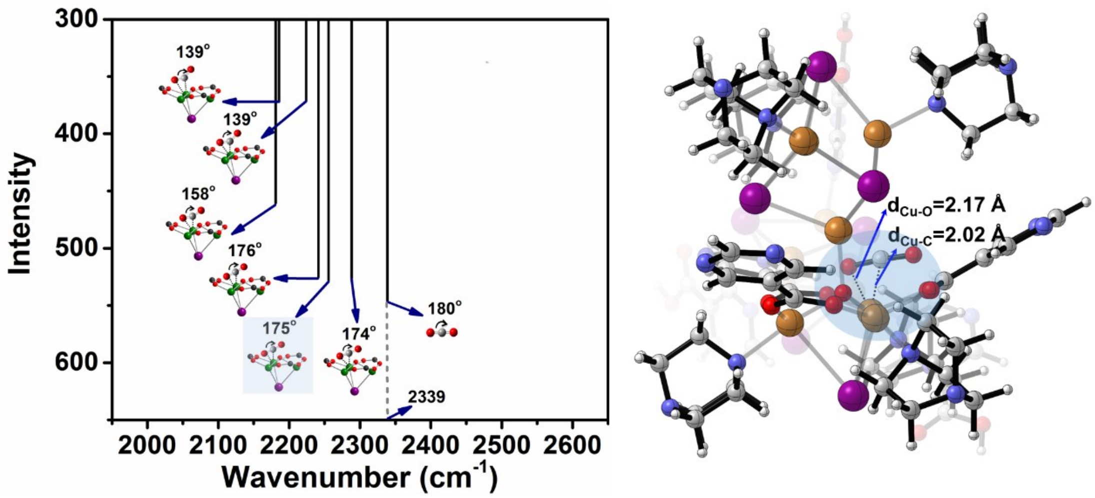

Figure S34. DFT predicted in situ FTIR spectra of CO_2 adsorption on **NJU-Bai61**.

Table S9. The values of IR wavenumber and the geometry parameters in CO_2 of **NJU-Bai61** calculated by DFT.

Number	$d_{Cu\text{-}O}$ (Å)	$d_{Cu\text{-}C}$ (Å)	Angle (°)	Wavenumber[a] (cm^{-1})	Intensity
			Free CO_2		
1			180	2339	546
			adsorbed CO_2		
2	3.16	2.69	139	2224	371
3	3.17	2.69	139	2186	372
4	2.38	2.10	158	2181	461
5	2.19	2.03	176	2241	525
6	2.17	2.02	175	2256	528
7	2.19	2.02	174	2288	526

[a]scaled factor is 0.960.

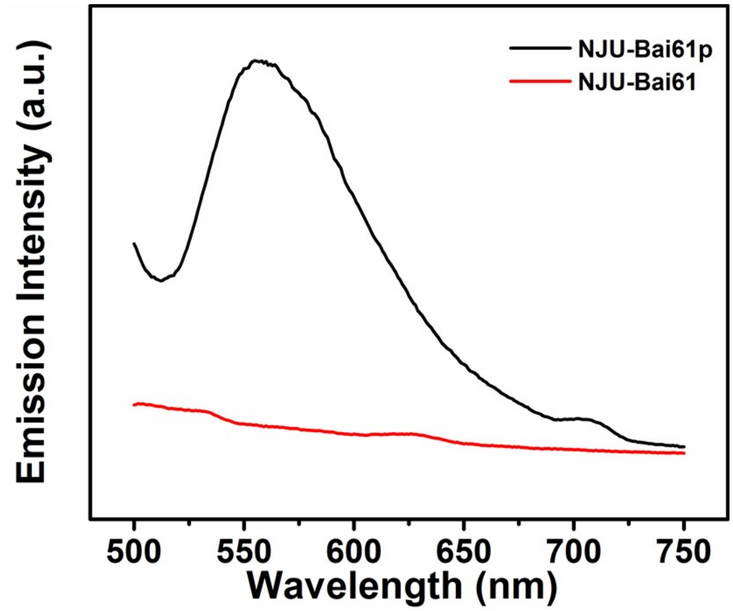

Figure S35. PL spectra of **NJU-Bai61p** and **NJU-Bai61**.

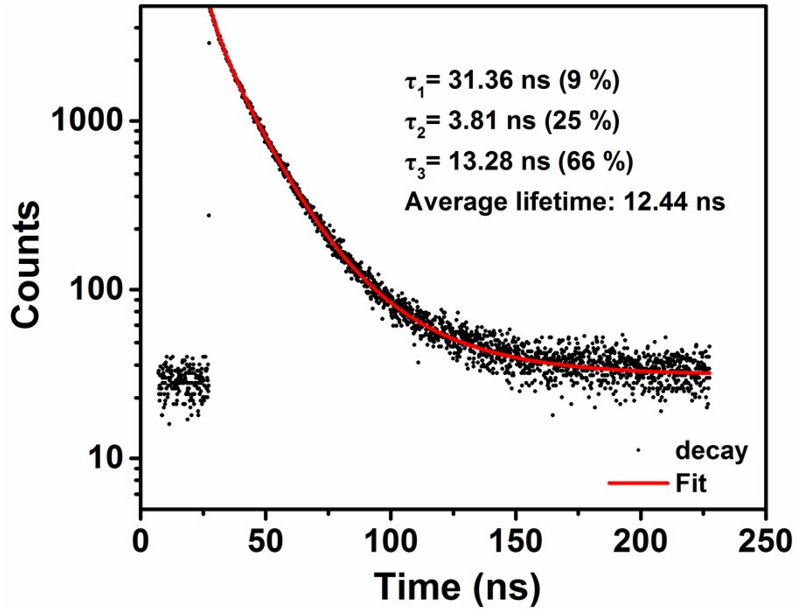

Figure S36. PL decay curve of **NJU-Bai61p**.

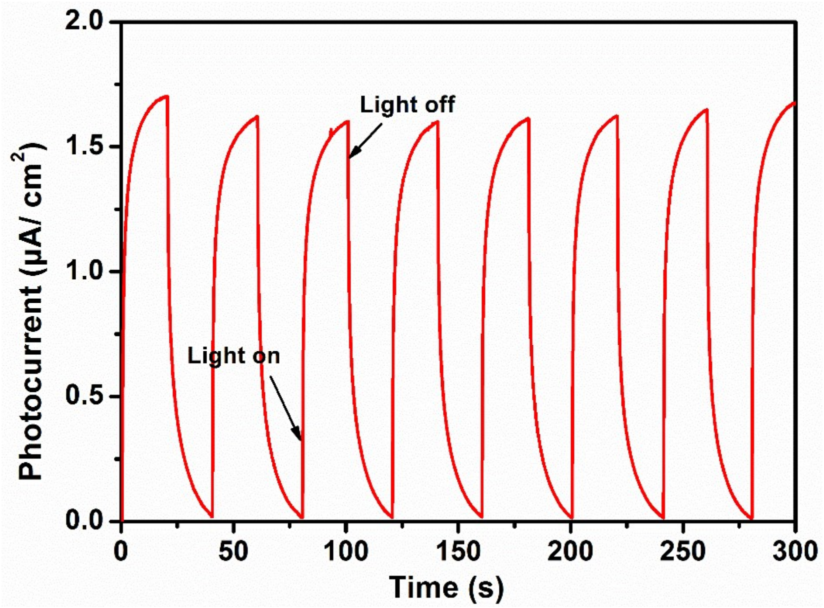

Figure S37. Transient photocurrent response of **NJU-Bai61**.

The transient photocurrent curve of **NJU-Bai61** for several on-off cycles under light irradiation presented rapid, stable and invertible photocurrent response (1.7 μA/cm^2), which are also favorable for driving the photocatalytic process.

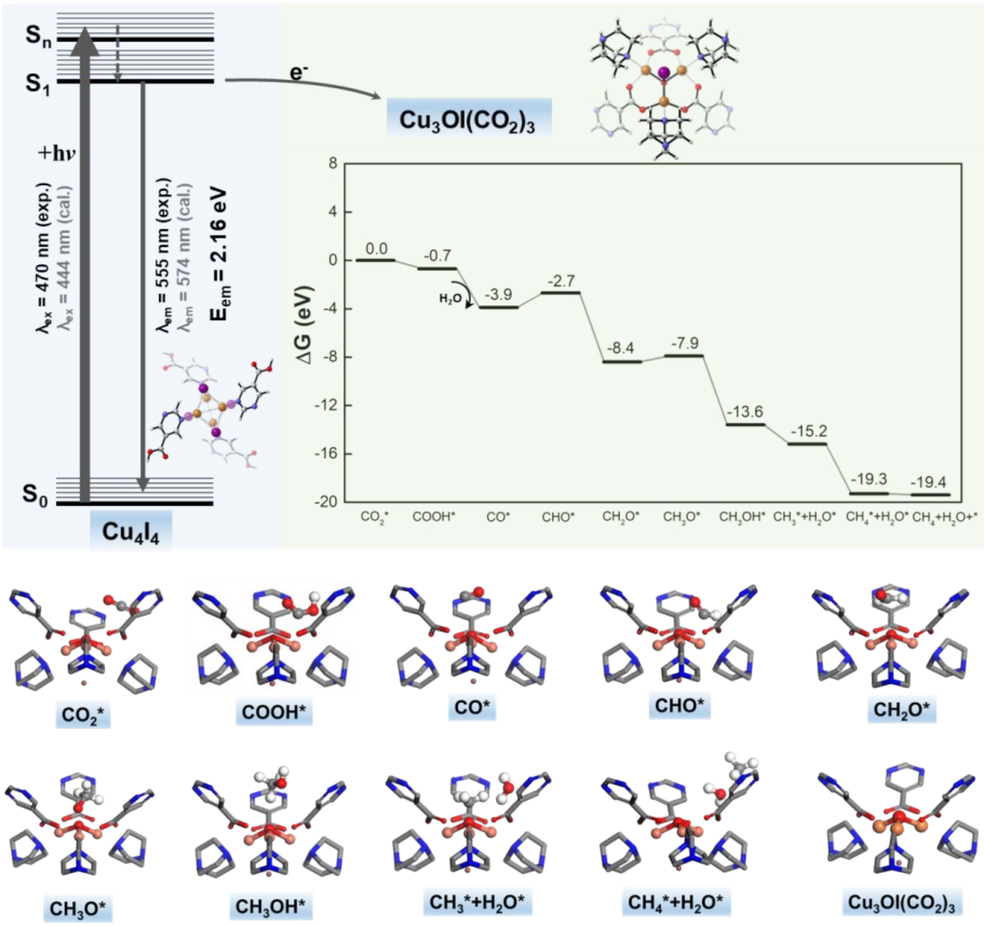

Figure S38. A possible reaction pathway for the CO_2 photoreduction to CH_4 over **NJU-Bai61**.

The 470 nm (440 nm calculated by TDDFT) light launches an electronic transition from the ground state (S_0) to a certain excited state (S_n) in Cu_4I_4 cluster, followed by quick deactivation processes to the first excited state (S_1). TDDFT calculations predicted the emission at 574 nm, which is in agreement with the experiment of 555 nm. The electron may be transferred to the $Cu_3OI(CO_2)_3$ cluster, while the $Cu_3OI(CO_2)_3$ clusters could supply electrons to the adsorbed CO_2 for CH_4 evolution. In the first step, the adsorbed CO_2 molecule is reduced to the COOH* species with releasing 0.7 eV of free energy. Further the COOH* combines with the electron-proton pair to generate CO* with the ΔG of -3.2 eV. Then CO* accepts two electrons and a proton to form CHO*, which is endothermic process with the ΔG of 1.2 eV. The Cu_4I_4 cluster could serve as a photosensitizer and donate the energy of 2.16 eV to the process of CO* to CHO* at the $Cu_3OI(CO_2)_3$ cluster. The CHO* protonates to form CH_2O* (ΔG = -5.7 eV). Similarly, two electrons are transferred together with a proton to the CH_2O* to form CH_3O*, then combining with a proton to obtain CH_3OH*. The ΔG values of these two steps are 0.5 and -5.7 eV, respectively. The CH_3OH* is reduced with two electrons and one proton to generate CH_3* and H_2O* (ΔG = -1.6 eV). A proton is subsequently transferred to the CH_3* to generate CH_4*, which the ΔG is -4.1 eV. The catalytic cycle is completed after release of CH_4 and H_2O molecules calculated by -0.1 eV.

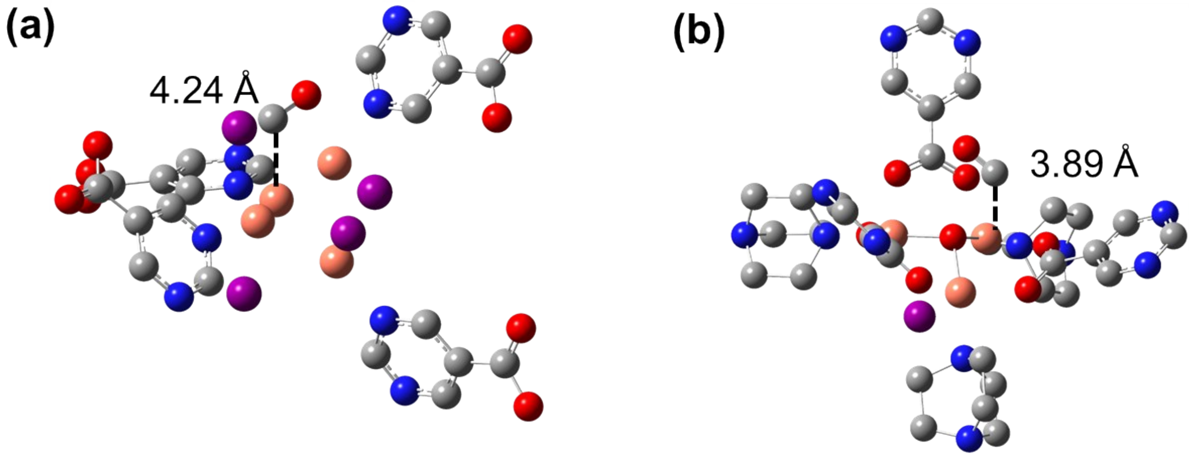

Figure S39. The CO binding structures on **NJU-Bai61p** (a) and **NJU-Bai61** (b).

References

[1] K. Baishya, J. S. Ray, P. Dutta, P. P. Das and S. K. Das, *Appl. Phys. A-Mater.,* 2018, **124**, 704.

[2] a) K. S. Walton and R. Q. Snurr, *J. Am. Chem. Soc.,* 2007, **129**, 8552; b) J. Rouquerol, P. Llewellyn and F. Rouquerol, *Stud. Surf. Sci. Catal.,* 2007, **160**, 49.

[3] J. L. C. Rowsell and O. M. Yaghi, *J. Am. Chem. Soc.,* 2006, **128**, 1304.

[4] M. J. Frisch, G. W. Trucks, H. B. Schlegel, G. E. Scuseria, M. A. Robb, J. R. Cheeseman, G. Scalmani, V. Barone, G. Scalmani, V. Barone, G. A. Petersson, H. Nakatsuji, X. Li, M. Caricato, A. V. Marenich, J. Bloino, B. G. Janesko, R. Gomperts, B. Mennucci, H. P. Hratchian, J. V. Ortiz, A. F. Izmaylov, J. L. Sonnenberg, D. Williams-Young, F. Ding, F. Lipparini, F. Egidi, J. Goings, B. Peng, A. Petrone, T. Henderson, D. Ranasinghe, V. G. Zakrzewski, J. Gao, N. Rega, G. Zheng, W. Liang, M. Hada, M. Ehara, K. Toyota, R. Fukuda, J. Hasegawa, M. Ishida, T. Nakajima, Y. Honda, O. Kitao, H. Nakai, T. Vreven, K. Throssell, J. A. Montgomery, Jr., J. E. Peralta, F. Ogliaro, M. J. Bearpark, J. J. Heyd, E. N. Brothers, K. N. Kudin, V. N. Staroverov, T. A. Keith, R. Kobayashi, J. Normand, K. Raghavachari, A. P. Rendell, J. C. Burant, S. S. Iyengar, J. Tomasi, M. Cossi, J. M. Millam, M. Klene, C. Adamo, R. Cammi, J. W. Ochterski, R. L. Martin, K. Morokuma, O. Farkas, J. B. Foresman and D. J. Fox, Gaussian 16, Revision A.03, Gaussian, Inc., Wallingford, CT, 2016.

[5] A. D. Becke, *J. Chem. Phys.,* 1993, **98**, 5648.

[6] W. R. Wadt and P. J. Hay, *J. Chem. Phys.,* 1985, **82**, 284.

[7] M. D. Segall, P. J. D. Lindan, M. J. Probert, C. J. Pickard, P. J. Hasnip, S. J. Clark and M. C. Payne, *J. Phys.: Condens. Matter,* 2002, **14**, 2717.

[8] *Materials Studio,* version 7.0; Accelrys, Inc.: San Diego, CA, 2013.

[9] J. P. Perdew, K. Burke and M. Ernzerhof, *Phys. Rev. Lett.,* 1996, **77**, 3865.

[10] S. Grimme, J. Antony, S. Ehrlich and H. Krieg, *J. Chem. Phys.,* 2010, **132**, 154104.
[11] S. Grimme, S. Ehrlich and L. Goerigk, *J. Comput. Chem.,* 2011, **32**, 1456.
[12] G. M. Sheldrick, *Acta Crystallogr. Sect. A,* 2008, **64**, 112.
[13] A. L. Spek, *J. Appl.Crystallogr.,* 2003, **36**, 7.
[14] F. Yu, M. Kurmoo, G.-L. Zhuang and J.-L. Zuo, *Chem. Sci.,* 2018, **9**, 7498.
[15] H. Zhang, J. Wei, J. Dong, G. Liu, L. Shi, P. An, G. Zhao, J. Kong, X. Wang, X. Meng, J. Zhang and J. Ye, *Angew. Chem. Int. Ed.,* 2016, **55**, 14310.
[16] E. Chen, M. Qiu, Y.-F. Zhang, Y.-S. Zhu, L.-Y. Liu, Y.-Y. Sun, X. Bu, J. Zhang and Q. Lin, *Adv. Mater.,* 2018, **30**, 1704388.
[17] G. Xu, H. Zhang, J. Wei, H.-X. Zhang, X. Wu, Y. Li, C. Li, J. Zhang and J. Ye, *ACS Nano,* 2018, **12**, 5333.
[18] S. Wan, M. Ou, Q. Zhong and X. Wang, *Chem. Eng. J.,* 2019, **358**, 1287.
[19] L.-Y. Wu, Y.-F. Mu, X.-X. Guo, W. Zhang, Z.-M. Zhang, M. Zhang and T.-B. Lu, *Angew. Chem. Int. Ed.,* 2019, **58**, 9491.
[20] Z.-C. Kong, J.-F. Liao, Y.-J. Dong, Y.-F. Xu, H.-Y. Chen, D.-B. Kuang and C.-Y. Su, *ACS Energy Lett.,* 2018, **3**, 2656.
[21] S. Yan, S. Quyang, H. Xu, M. Zhao, X. Zhang and J. Ye, *J. Mater. Chem. A,* 2016, **4**, 15126.
[22] R. Li, J. Hu, M. Deng, H. Wang, X. Wang, Y. Hu, H.-L. Jiang, J. Jiang, Q. Zhang, Y. Xie and Y. Xiong, *Adv. Mater.,* 2014, *2***6**, 4783.
[23] X. He and W.-N. Wang, *J. Mater. Chem. A,* 2018, **6**, 932.
[24] M. Wang, D. Wang and Z. Li, *Appl. Catal. B-Environ.,* 2016, **183**, 47.
[25] F. Guo, S. Yang, Yi. Liu, P. Wang, J. Huang and W.-Y. Sun, *ACS Catal.,* 2019, **9**, 8464.
